# Supplementary material for: A universal test for gravitational decoherence
Source: Nat Commun. 2016 Oct 3;7:13022. doi: 10.1038/ncomms13022 (PMC5063961; doi:10.1038/ncomms13022)
Supplement: Supplementary Information — Supplementary Figures 1-15, Supplementary Notes 1-6 and Supplementary References. [file ncomms13022-s1.pdf]

Supplementary Information

Supplementary Figures

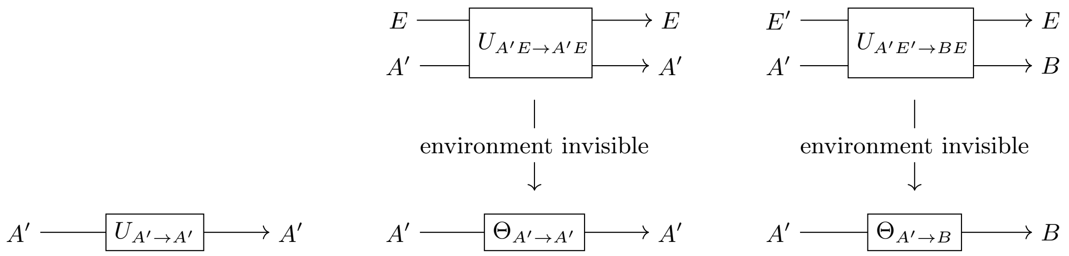

(a) Closed system

(b) Open system, fixed visible part

(c) Open system, changing visible part

Supplementary Fig. 1: **Different kinds of dynamical evolution.** (a) If a system A' evolves as a closed system, its state transformation is described by a unitary $U_{A' \to A'}$. (b) If it evolves in interaction with another system E that is not part of our description, its state transformation is described by a TPCPM $\Theta_{A' \to A'}$. (c) If the visible part of the overall system before and after the evolution is not the same, we describe the state transformation by a TPCPM $\Theta_{A' \to B}$.

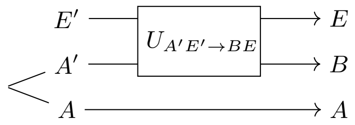

Supplementary Fig. 2: **Purification to three systems.** A lot of useful insight into the channel can be gained by considering a system A that is entangled with the input system A' of the channel. This leads to a situation with three systems A, B and E, controlled by Alice, Bob and Eve.

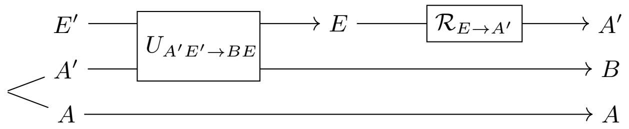

Supplementary Fig. 3: **Overall picture of our decoherence analysis for quantum theory.** We interpret $H_{\min}(A|E)_\rho$ as a measure for how close Eve can get to maximal entanglement with Alice by applying a transformation $\mathcal{R}_{E \to A'}$ on her share of the state ρ_{ABE}.

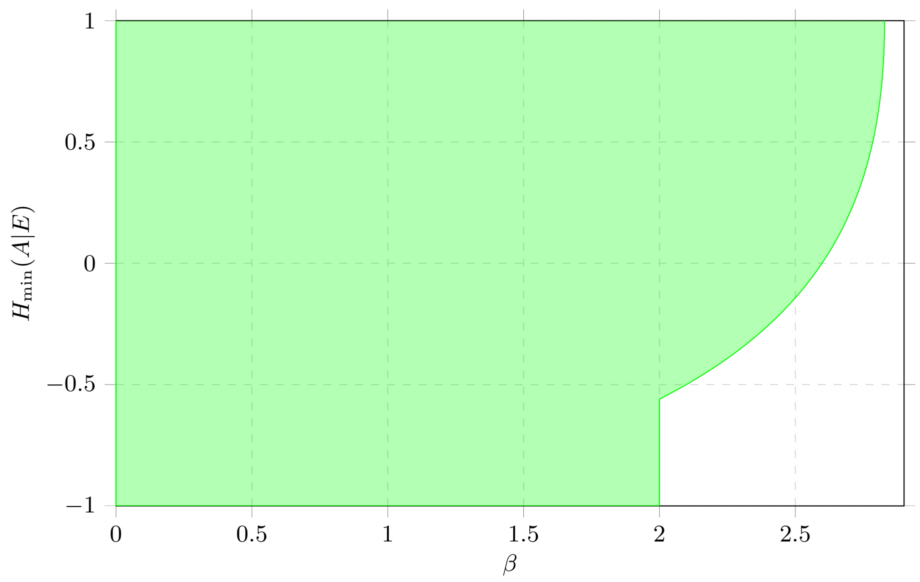

Supplementary Fig. 4: **The feasible region for the min-entropy in the qubit case.** If a point with coordinates (x, y) is colored in green, then there exists a quantum state ρ_{ABE}, where A and B are qubit systems, and observables A_0, A_1, B_0, B_1 such that $\beta = x$ and $H_{\min}(A|E) = y$. The green region is tight, i.e. for the white region in this figure, there is no combination of state and observables achieving the corresponding values of β and $H_{\min}(A|E)$.

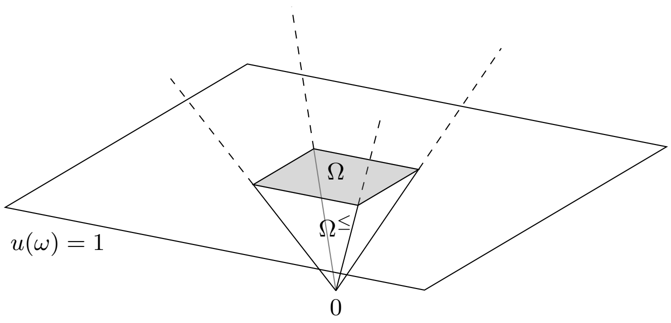

Supplementary Fig. 5: **Visualization of an abstract state space.** The set V^+ is a cone in a vector space V, here shown as a cone with a square base. The normalized states Ω are given by the intersection of V^+ with the plane on which the functional u takes the value 1. The set $\Omega^{\leqslant}$ consists of those elements of V^+ on which the functional u takes values between 0 and 1.

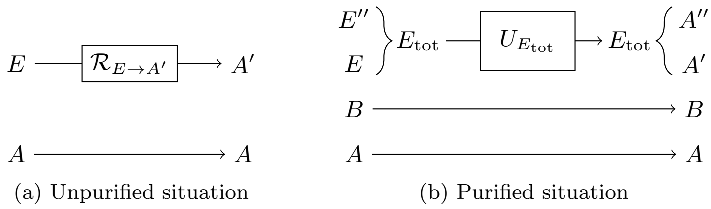

Supplementary Fig. 6: **Purification of equation (14).** Part (a) shows the system and maps involved in expression (14) for the quantum min-entropy. In expression (92), we purify this situation, as shown in (b), to arrive at a situation with three parties A, B and E_{tot}, and with a map $U_{E_{\mathrm{tot}}}$ which acts on one system E_{tot} alone rather than mapping from one system to another.

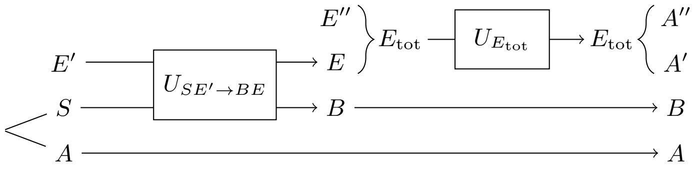

Supplementary Fig. 7: **Overall picture of our decoherence analysis for GPTs.** Since the purifying system B in expression (92) is not specified, we can choose it such that it fits our situation for the decoherence analysis.

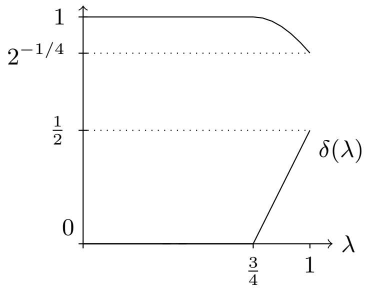

Supplementary Fig. 8: **Plot of the result.** The bound $2^{-\delta^2(\lambda)}$ is non-trivial precisely when the CHSH winning probability for Alice and Bob is non-classical, i.e. $\lambda > 3/4$.

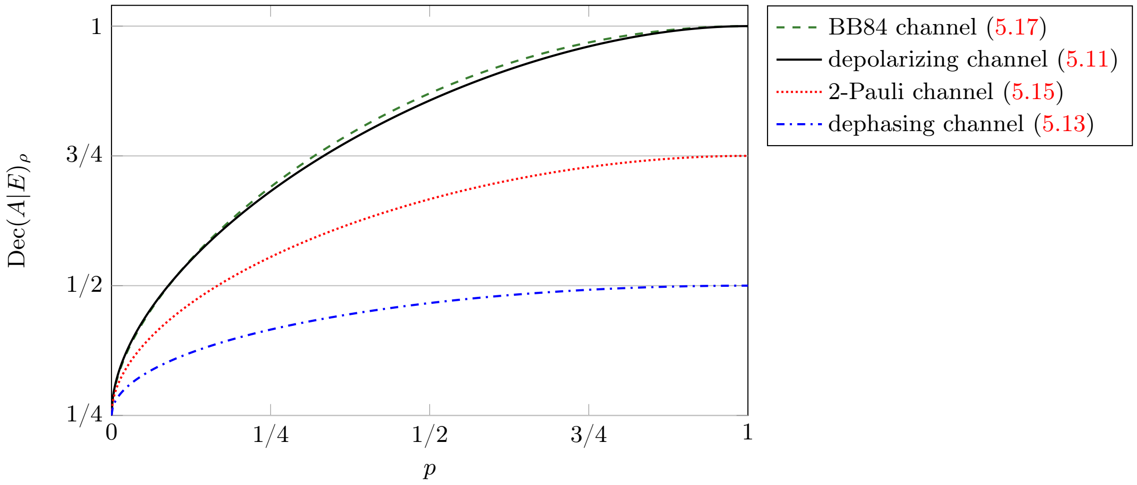

Supplementary Fig. 9: **Values of the decoherence quantity for some Pauli channels.** The strength of the shown Pauli channels depends on the number of Pauli operators that are involved. The dephasing channel, with one involved Pauli operator, achieves a decoherence of 1/2, the 2-Pauli channel achieves a decoherence of 3/4, and the depolarizing channel and the BB84 channel (with three involved Pauli operators) achieve a decoherence of 1.

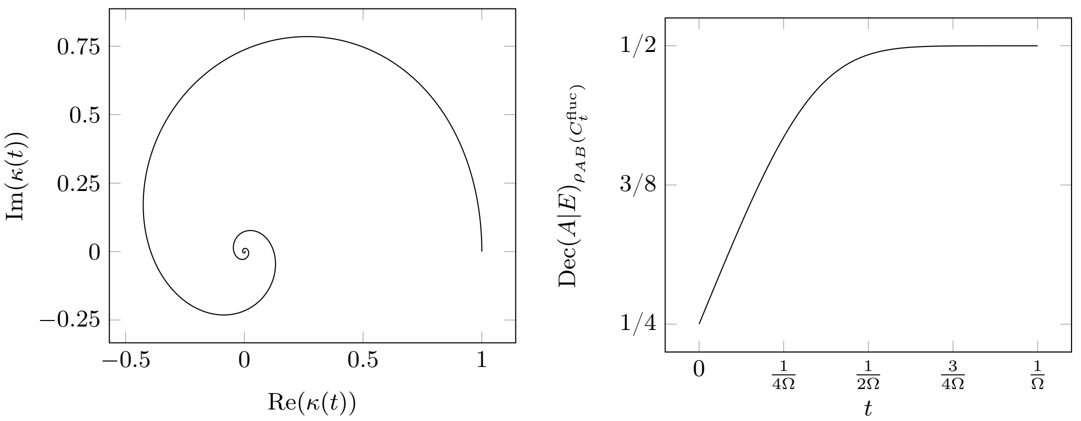

Supplementary Fig. 10: **Plots for gravitational decoherence due to flat spacetime fluctuations.** On the left, the off-diagonal element $\kappa(t)$, equation (200), of the Choi matrix (192) is shown as a parametric plot for $0 \leqslant t \leqslant 1/\Omega$. The inward spiralling is a consequence of the phase rotation $\alpha(t)$, together with the exponential decay of the entry's absolute value. On the right, the decoherence quantity (214) of the channel is plotted as a function of the time t. It is a dephasing channel with a deformed strength parameter p. The plots have been made for the parameters $v = 9/10$, $\sigma = 3/2$, which we have chosen purely for illustration purposes. The analysis presented here is valid for any such parameters.

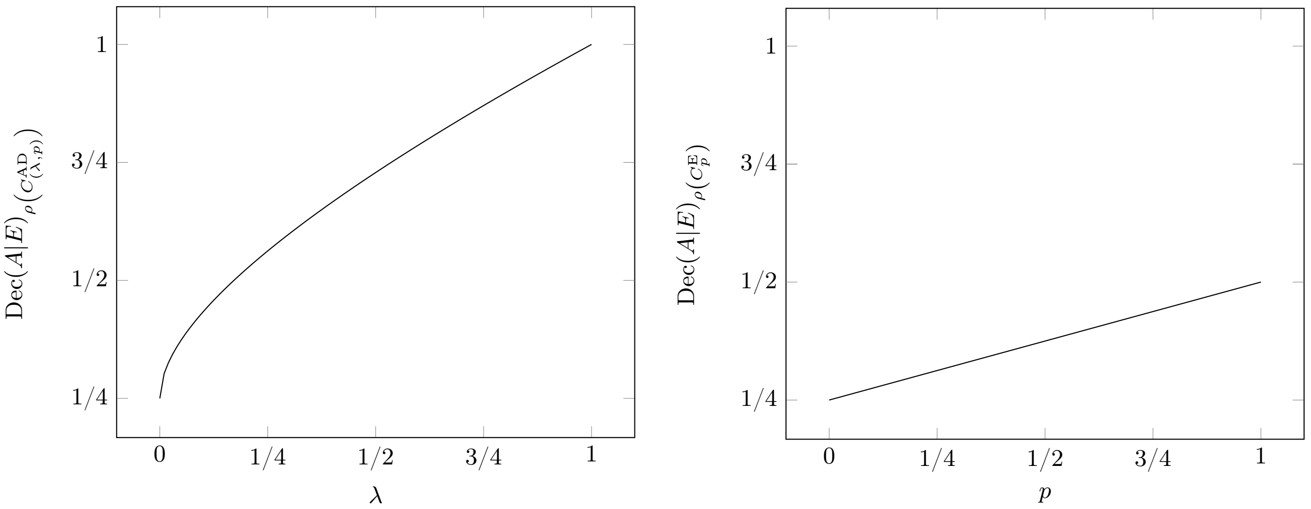

Supplementary Fig. 11: **Values of the decoherence quantity for the amplitude damping channel and the erasure channel.** On the left hand side, a plot of the decoherence quantity of the amplitude damping channel is shown. It achieves full decoherence for $\lambda = 1$. In that maximally decoherent case, the channel simply replaces any input state by the $|0\rangle$ state. The erasure channel, in contrast, replaces any input state by the orthogonal $|2\rangle$ state for $p = 1$. As the plot on the right hand side shows, this decoheres the system much less, as measured by our decoherence quantity.

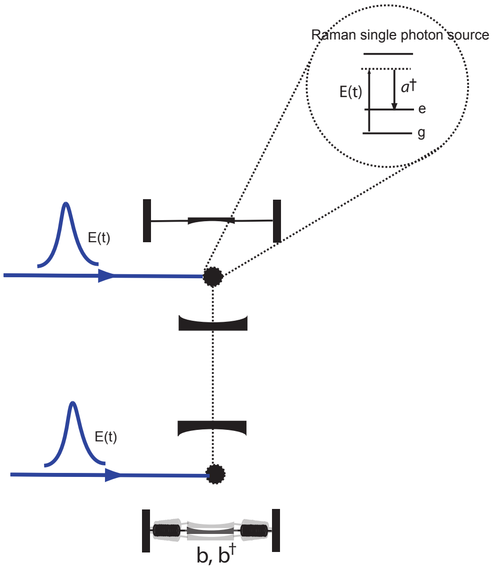

Supplementary Fig. 12: **Illustration of the optomechanical setup.** Two cavities each contain a Raman single photon source controlled by an external laser 'write field' $E(t)$. The Raman sources are first prepared in an entangled state. Only one cavity contains a mechanical element coupled by radiation rouser to the cavity field.

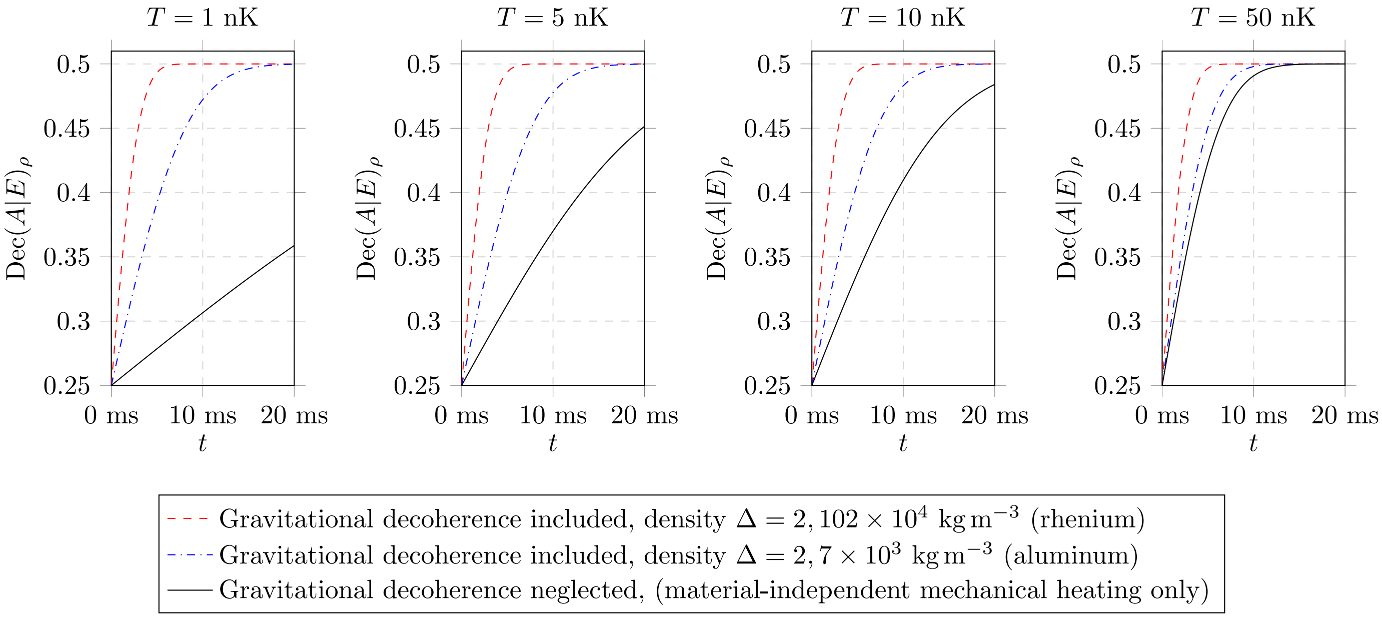

Supplementary Fig. 13: **Predicted values of the decoherence quantity in the optomechanical experiment.** The decoherence quantity $\mathrm{Dec}(A|E)_\rho$ as in Equation (250) is plotted as a function of time for different temperatures and two different materials of the mechanical element. In addition, $\mathrm{Dec}(A|E)_\rho$ is plotted for the case where there is no gravitational decoherence, Equation (253). The calculations have been made for the experimental parameters $g_0 = 1$ s^{-1}, $\omega_m = 1$ s^{-1} and $\gamma_m = 10^{-10}$ s^{-1}.

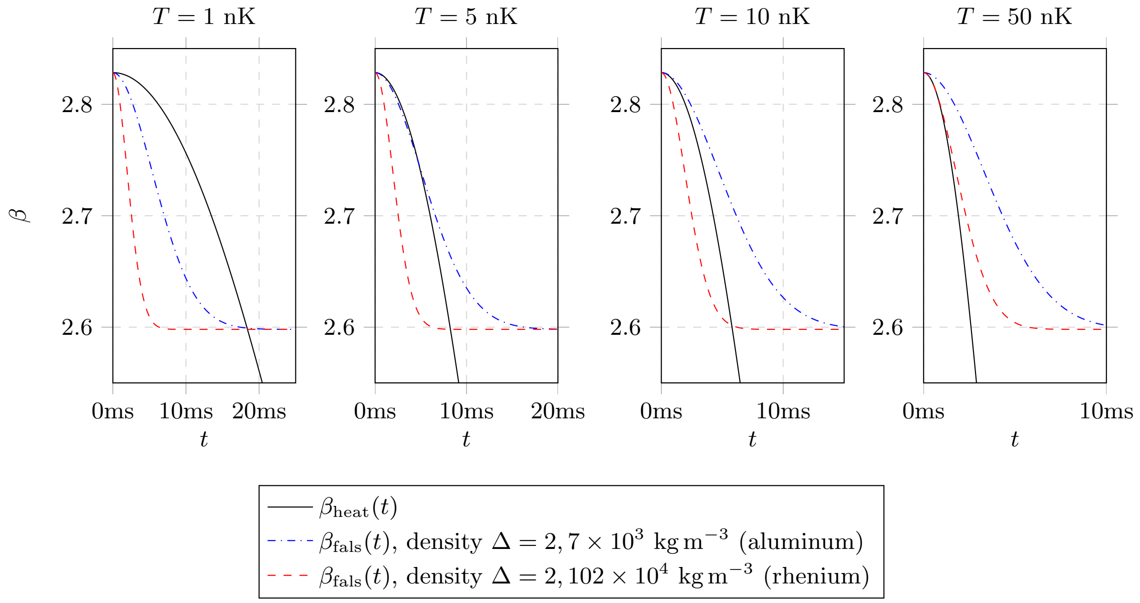

Supplementary Fig. 14: **Minimal CHSH values for the falsification of the gravitational decoherence model.** The quantity β_{fals}, which is the minimal value that needs to be exceeded in the measurement of the CHSH value β in order to rule out the gravitational decoherence model, is plotted as a function of time for the same materials and temperatures as above. In addition, the value β_{mech} is plotted, which is the CHSH value that can actually be measured using the standard CHSH measurement in the case where gravitational decoherence is absent and only mechanical heating contributes to the decoherence.

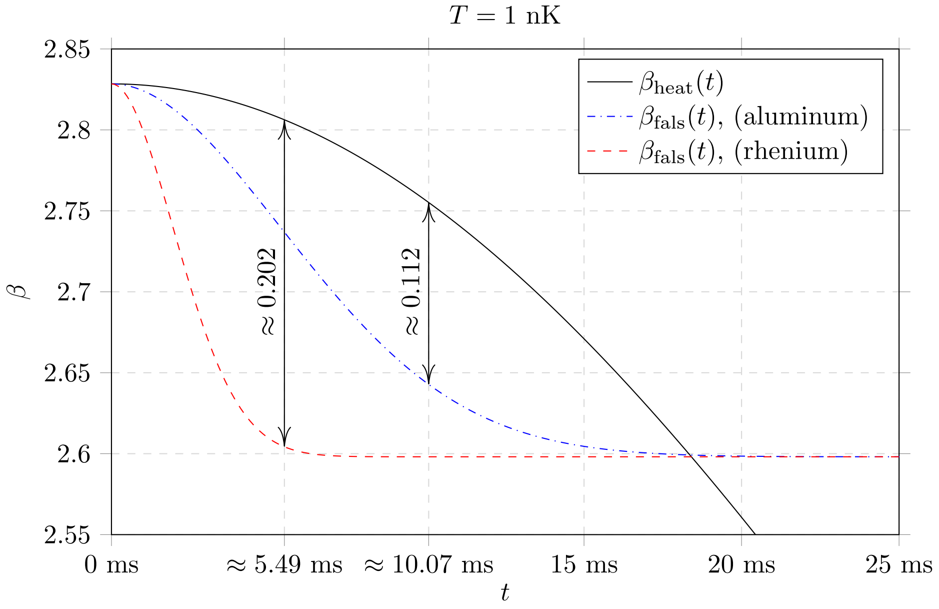

Supplementary Fig. 15: **Optimal measurement times for ruling out the gravitational decoherence model.** The three plots are identical to the ones in the leftmost box in Supplementary Fig. 14, i.e. for $T = 1$ nK. In addition, the time t_{max} at which the gap $g(t)$ between β_{mech} and β_{fals} is maximal is indicated for the two cases where the material of the mechanical element has the density of aluminum or rhenium.

Supplementary Note 1: Background – Decoherence in quantum theory

In this appendix, we give a short introduction to decoherence in quantum theory. It consists of concepts, results and quantities that are well-established in quantum information science [1]. The topics are chosen to facilitate the understanding of our contributions in Supplementary Notes 2 and 3 rather than to give a full introduction to the subject of decoherence. In the first subsection, we explain that the dynamical evolution (e.g. decoherence) can equivalently be treated in a picture where the system is entangled with another system. The purification of that system after the evolution is a tripartite system. This tripartite state plays a central role in our later analysis. We emphasize that working in this picture is not a loss of generality but merely a picture that is equivalent to the single-system treatment, but which is more suitable for our analysis [2]. In the second subsection, we explain why the min-entropy is the relevant quantity in the information theoretic analysis of decoherence. The min-entropy is the quantity that we use for our analysis in Supplementary Note 2. It is also the quantity that serves as our motivation to define a decoherence quantity for generalized probabilistic theories in Supplementary Note 3. We note that a generalization of quantum theory by, for example, introducing additional terms into the Schrödinger equation fall under the regime of generalized theories in our discussion.

For this document, we make the following conventions.

- The logarithm is with respect to base 2, i.e. $\log \equiv \log_2$.
- Hilbert spaces are assumed to be finite-dimensional, unless otherwise stated.
- We denote the set of density operators (states) on a Hilbert space $\mathcal{H}$ by $\mathcal{S}(\mathcal{H})$.
- We identify operators on Hilbert spaces with their reordered versions resulting from permutations of systems. For example, for Hilbert spaces $\mathcal{H}_A$, $\mathcal{H}_B$, $\mathcal{H}_E$ and states $\Phi_{AE} \in \mathcal{S}(\mathcal{H}_A \otimes \mathcal{H}_E)$, $\sigma_B \in \mathcal{S}(\mathcal{H}_B)$, we identify the state $\Phi_{AE} \otimes \sigma_B \in \mathcal{S}(\mathcal{H}_A \otimes \mathcal{H}_E \otimes \mathcal{H}_B)$ with the state in $\mathcal{S}(\mathcal{H}_A \otimes \mathcal{H}_B \otimes \mathcal{H}_E)$ resulting from the application of the braiding map $\mathcal{H}_A \otimes \mathcal{H}_E \otimes \mathcal{H}_B \to \mathcal{H}_A \otimes \mathcal{H}_B \otimes \mathcal{H}_E$ on $\Phi_{AE} \otimes \sigma_B$.
- For a state $\rho_{ABE} \in \mathcal{S}(\mathcal{H}_A \otimes \mathcal{H}_B \otimes \mathcal{H}_E)$, we denote its reduced states by according changes of the subscript, e.g. $\rho_A := \mathrm{Tr}_B(\rho_{AE})$, $\rho_A := \mathrm{Tr}_B(\mathrm{Tr}_E(\rho_{ABE}))$.
- For a state $\rho_{ABE} \in \mathcal{S}(\mathcal{H}_A \otimes \mathcal{H}_B \otimes \mathcal{H}_E)$, entropies are evaluated for the according reduced states, e.g. $H(A|B)_\rho$ is the conditional von Neumann entropy of $\rho_{AB} = \mathrm{Tr}_E(\rho_{ABE})$ (c.f. Supplementary Note 1).

Dynamical evolution and its purification

Interaction and non-unitary evolution: Suppose that a system[1] A', initially in a state described by a density operator $\rho_{A'} \in \mathcal{S}(\mathcal{H}_{A'})$, undergoes a dynamical evolution over some time interval. If A' undergoes this evolution as a closed system, then according to one of the postulates of quantum mechanics, the state transforms as

$$\rho_{A'} \mapsto U_{A' \to A'}\, \rho_{A'}\, U^\dagger_{A' \to A'} \tag{1}$$

for a unitary $U_{A' \to A'} : \mathcal{H}_{A'} \to \mathcal{H}_{A'}$ (see Supplementary Fig. 1 (a)). In general, however, the system A' may be open, i.e. it may interact with another system E that is called the *environment*. We consider the environment E to consist of all the systems that interact with system A'. Taken together, the combined system $A'E$ then forms a closed system and hence evolves as

$$\rho_{A'} \otimes \rho_E \mapsto U_{A'E \to A'E}(\rho_{A'} \otimes \rho_E) U^\dagger_{A'E \to A'E}\,, \tag{2}$$

where ρ_E is the initial state of the environment and $U_{A'E \to A'E} : \mathcal{H}_{A'} \otimes \mathcal{H}_E \to \mathcal{H}_{A'} \otimes \mathcal{H}_E$ is a unitary.

We may be ignorant about the environment E and only have access to system A'. Our description would then treat the state of the subsystem A' after the evolution as a function of the state $\rho_{A'}$ of A' before the evolution. We arrive at this description by taking the partial trace over E in expression (2):

$$\rho_{A'} \mapsto \mathrm{Tr}_E \left(U_{A'E \to A'E}(\rho_{A'} \otimes \rho_E) U^\dagger_{A'E \to A'E} \right) =: \Theta_{A' \to A'}(\rho_{A'})\,. \tag{3}$$

---

[1] Here we denote the system by A' rather than A because we reserve the label A for another system (see Supplementary Fig. 2 and 3). This way, the labeling convention of the main article coincides with the labeling in the Supplementary Information.

A map $\Theta_{A'\to A'}$ of the form (3) is easily shown to be a trace-preserving completely positive map (TPCPM). Thus, the evolution of an open system A', when the environment E is not visible, is described by a TPCPM $\Theta_{A'\to A'}$ (see Supplementary Fig. 1 (b)).

In a yet more general case, it may be that after the evolution of the system $A'E$, we do not have access to system A' but to a different subsystem B of $A'E$. An example would be a two-particle system A' interacting with another two-particle system, where we only have access to one particle (B) of the four particles after the evolution. Mathematically speaking, the fact that we see a different subsystem before and after the evolution means that our factorization of the overall Hilbert space changes: Before the evolution, we write $\mathcal{H} = \mathcal{H}_{A'} \otimes \mathcal{H}_{E'}$ and after the evolution, we write $\mathcal{H} = \mathcal{H}_B \otimes \mathcal{H}_E$ (see Supplementary Fig. 1 (c)). Thus, the unitary evolution of the closed overall system is described by a unitary $U_{A'E'\to BE} : \mathcal{H}_{A'} \otimes \mathcal{H}_{E'} \to \mathcal{H}_B \otimes \mathcal{H}_E$. Describing only the accessible part before and after the evolution, we end up with a TPCPM

$$\rho_{A'} \mapsto \mathsf{Tr}_E \left(U_{A'E'\to BE}(\rho_{A'} \otimes \rho_{E'}) U^\dagger_{A'E'\to BE} \right) =: \Theta_{A'\to B}(\rho_{A'}) \,. \tag{4}$$

Thus, the evolution of a system A' to a system B, when being ignorant about the environment, is described by a TPCPM $\Theta_{A'\to B}$.

Stinespring dilation: We have demonstrated that unitaries on two (or more) systems give rise to TPCPMs on one system. It is well-known that the converse is also true: Every TPCPM $\Theta_{A'\to B} : \mathcal{S}(\mathcal{H}_{A'}) \to \mathcal{S}(\mathcal{H}_B)$ can be extended to a unitary $U_{A'E'\to BE}$ on a larger system in the following sense. For Hilbert spaces $\mathcal{H}_{E'}$ and $\mathcal{H}_E$ of appropriate dimensions, it holds that for every pure state $|0\rangle\langle 0|_{E'}$ on $\mathcal{H}_{E'}$, there is a unitary

$$U_{A'E'\to BE} : \mathcal{H}_{A'} \otimes \mathcal{H}_{E'} \to \mathcal{H}_B \otimes \mathcal{H}_E \tag{5}$$

such that

$$\mathsf{Tr}_E(U_{A'E'\to BE}(\rho_{A'} \otimes |0\rangle\langle 0|_{E'}) U^\dagger_{A'E'\to BE}) = \Theta_{A'\to B}(\rho_{A'}) \quad \text{for all } \rho_{A'} \in \mathcal{S}(\mathcal{H}_{A'}) \,. \tag{6}$$

This (or an equivalent statement) is the *Stinespring dilation theorem* [3]. For more details see [1].

Textbook definitions of decoherence: From now on, we will take the viewpoint that the TPCPM $\Theta_{A'\to B}$ is what we are given in the first place. Physically speaking, we assume that we are in the setting where all that we observe is a process in which a system A' in some state $\rho_{A'}$ transforms into a state ρ_B of some system B. We think of this as a *channel* $\Theta_{A'\to A'}$, into which we input a system A' and get a system B as an output. Our goal in this Supplementary Note is to find a precise mathematical formulation of the following question in the quantum theoretical framework: How much does the channel *decohere* the system?

For the case where $A' = B$, the standard quantum mechanics literature gives some simple descriptions of what the decoherence of a system under a dynamical evolution is. As an example, consider the case where A' is a spin-1/2 particle, initially in the spin "up" state in the x-direction,

$$\rho_{A'} = |\uparrow_x\rangle\langle\uparrow_x| \,, \quad |\uparrow_x\rangle = \frac{|\uparrow_z\rangle + |\downarrow_z\rangle}{\sqrt{2}} \,. \tag{7}$$

If the channel $\Theta_{A'\to A'}$ is given by a measurement of the spin in the z-direction, then, written in the z-basis, the state of the system transforms as

$$\rho_{A'} = \begin{pmatrix} 1/2 & 1/2 \\ 1/2 & 1/2 \end{pmatrix} \mapsto \begin{pmatrix} 1/2 & 0 \\ 0 & 1/2 \end{pmatrix} = \Theta_{A'\to A'}(\rho_{A'}) \,. \tag{8}$$

One possible observation one can make in (8) is that the spin measurement in the z-direction causes the off-diagonal terms of the density matrix to vanish. This is an extreme case of the *dephasing channel* in the z-basis, which causes a loss of the phase information of the superposition (7). This loss of phase information is often equated with decoherence. Another feature of (8) that is often said to be the characteristic of decoherence is that $\Theta_{A'\to A'}$ turns an initially pure state into a mixed state.

These descriptions of decoherence, valid in their own right, are not favored by us for mainly three reasons. Firstly, these are no quantitative measures of decoherence. Secondly, they lack a clear operational meaning. Thirdly, they rely on the quantum mechanical formalism, in which states are expressed as density operators. It is not clear how to express them in more general cases that are not described by quantum theory.

The min-entropy as a measure for decoherence

A lot can be learned about the channel if one takes an additional system into the picture. From now on, we consider the case where the input system A' is purified by a system A. Then, while system A' undergoes the channel evolution, system A remains unchanged. For example, one such case is the case where the system AA' is in a maximally entangled state. This leads to the situation shown in Supplementary Fig. 2. The output system is now a tripartite system ABE. In quantum information science, it is very popular to think of the systems as being controlled by *parties* with intentions and interests rather than just being dead physical objects. We will follow this spirit and from now on use the language of a game and speak of parties Alice, Bob and Eve, that we think of as agents controlling the systems A, B and E. In quantum information science, it has been realized that important quantitative measures of the channel are functions of this tripartite state ρ_{ABE}.

The coherent information: One such measure quantifying decoherence is the *coherent information* [4]. It is defined in terms of the conditional von Neumann entropy

$$H(A|B)_\rho := H(AB)_\rho - H(B)_\rho \,, \tag{9}$$

where $H(AB)_\rho = -\mathsf{Tr}(\rho_{AB}\log(\rho_{AB}))$ and $H(B) = -\mathsf{Tr}(\rho_B \log(\rho_B))$ is the von Neumann entropy of the reduced state ρ_{AB} and ρ_B, respectively. The coherent information is defined as

$$I(A\rangle B)_\rho := -H(A|B)_\rho \,. \tag{10}$$

The coherent information $I(A\rangle B)_\rho$ has been shown to be related to the quantum channel capacity $Q(\Theta_{A'\to B})$ of $\Theta_{A'\to B}$, which is known as the Lloyd-Shor-Devetak (LSD) theorem [5–7]. It says that

$$Q(\Theta_{A'\to B}) = \lim_{n\to\infty} \frac{1}{n} \max_{\rho_{(A')^n}\in\mathcal{S}(\mathcal{H}_{A'}^{\otimes n})} I(A^n\rangle B^n)_\rho \,, \tag{11}$$

where $I(A^n\rangle B^n)_\rho$ is the coherent information for $\rho_{A^nB^n} = \mathbb{1}_A^{\otimes n} \otimes \Theta_{A'\to B}^{\otimes n}(\rho_{A^n(A')^n})$ and $\rho_{A^n(A')^n}$ is a purification of $\rho_{(A')^n}$. The state $\mathbb{1}_A^{\otimes n} \otimes \Theta_{A'\to B}^{\otimes n}(\rho_{A^n(A')^n})$ results from the n-fold use of the channel $\Theta_{A'\to B}$ to transmit $(A')^n$, i.e. n copies of system A', while the purification A^n of $(A')^n$ remains unchanged. Thus, the r.h.s. of (11) is the coherent information in the limit of infinitely many channel uses. Likewise, the quantum capacity $Q(\Theta_{A'\to B})$ is the limit of the achievable rate for quantum data transmission in the limit of infinitely many channel uses. One says that the quantum capacity, and therefore the coherent information, is an *asymptotic quantity*. This has the disadvantage that from the coherent information, only very limited statements about finitely many uses of the channel can be made.

The min-entropy: More insight about the behavior of the channel under finitely many uses can be gained by considering the corresponding *single-shot* quantity. To formulate it, note that the state ρ_{ABE} is pure, in which case the duality relation $H(A|B)_\rho = -H(A|E)_\rho$ for the conditional von Neumann entropy holds. This gives us

$$I(A\rangle B)_\rho = H(A|E)_\rho \,. \tag{12}$$

The corresponding single-shot quantity for the conditional von Neumann entropy $H(A|E)_\rho$ is the *conditional min-entropy*, or just *min-entropy*, $H_{\min}(A|E)_\rho$ [8]. It is defined as

$$H_{\min}(A|E)_\rho = \max_{\sigma_E} \sup\{\lambda \in \mathbb{R} \mid \rho_{AE} \leqslant 2^{-\lambda}\mathbb{1}_A \otimes \sigma_E\} \,, \tag{13}$$

where the maximum is taken over all subnormalized density operators on $\mathcal{H}_E$, i.e. all positive operators on $\mathcal{H}_E$ with trace between 0 and 1. The min-entropy quantifies the maximal size of a subsystem of A that can be decoupled from E [9], and thus tells us how many EPR pairs between Alice and Bob can be created [10] given a noisy output state ρ_{AB}. To obtain the single-shot capacity of n channel uses we are - as in the asymptotic case - allowed to optimize over input states $\rho_{A^n(A')^n}$. Clearly, however, the resulting expression can be lower bounded using a particular input state given by n copies of the maximally entangled state. This is the test state we employ here, and hence our test also provides a bound on the single shot capacity. For instance if A is a 2 level system, then the min-entropy readily quantifies the number of EPR-pairs we can recover, given that we started with n EPR pairs as an input. The min-entropy thus has a very appealing operational interpretation.

For our purposes, another expression for the min-entropy is more useful. In the following, we use the symbol $\simeq$ to denote that two Hilbert spaces are isomorphic, i.e. $\mathcal{H}_A \simeq \mathcal{H}_{A'}$ means that the two spaces have the same dimension. It has been shown [11] that the min-entropy can be expressed as

$$H_{\min}(A|E)_\rho = -\log d_A \max_{\mathcal{R}_{E\to A'}} F^2(\Phi_{AA'}, \mathbb{1}_A \otimes \mathcal{R}_{E\to A'}(\rho_{AE})) \,, \tag{14}$$

where d_A is the dimension of the Hilbert space $\mathcal{H}_A$ of system A, A' is a system with $\mathcal{H}_{A'} \simeq \mathcal{H}_A$, the maximization is carried out over all TPCPMs $\mathcal{R}_{E\to A'}$ from system E to system A', $F(\rho,\sigma) = \mathsf{Tr}\sqrt{\rho^{1/2}\sigma\rho^{1/2}}$ is the fidelity and $\Phi_{AA'}$ is a maximally entangled state on AA', i.e. $\Phi_{AA'}$ is an element of the set

$$\Gamma_{AA'} := \left\{ \Phi_{AA'} \in \mathcal{S}(\mathcal{H}_A \otimes \mathcal{H}_{A'}) \;\middle|\; \begin{array}{l} \text{There are bases } \{|i\rangle_A\}_i,\ \{|i\rangle_{A'}\}_i \text{ of } \mathcal{H}_A,\ \mathcal{H}_{A'} \text{ such that } \Phi_{AA'} = \\ |\phi\rangle\langle\phi|_{AA'} \text{ with } |\phi\rangle_{AA'} = \frac{1}{\sqrt{d_A}}\sum_i |i\rangle_A \otimes |i\rangle_{A'}. \end{array} \right\} . \tag{15}$$

The choice of $\Phi_{AA'} \in \Gamma_{AA'}$, i.e. the choice of bases for $\mathcal{H}_A$ and $\mathcal{H}_{A'}$, is irrelevant for the value of $H_{\min}(A|E)_\rho$. Since every $\Phi_{AA'} = |\phi\rangle\langle\phi|_{AA'} \in \Gamma_{AA'}$ is pure, we have that $F(\Phi_{AA'}, \sigma_{AA'}) = \sqrt{\langle\phi|\sigma|\phi\rangle_{AA'}}$ for any state $\sigma_{AA'}$ on AA'.

The expression (14) provides an intuition for the min-entropy. We think of the system ABE, which is in the pure state ρ_{ABE}, as being distributed between Alice, Bob and Eve. Imagine that Eve tries to perform operations on her share of the system with the intention to bring the reduced state between her and Alice as close as possible to the maximally entangled state $\Phi_{AA'}$, where the square of the fidelity is the measure of closeness. The closer Eve can bring the state to the maximally entangled state, the smaller the min-entropy $H_{\min}(A|E)_\rho$. The overall situation of our decoherence analysis is shown in Supplementary Fig. 3.

The min-entropy is strictly more informative than the conditional von Neumann entropy in the following sense. In the iid limit (which stands for *independent and identically distributed*), where many identically prepared systems go through the channel and end up in a state $\rho_{AB}^{\otimes n}$, the min-entropy converges to the conditional von Neumann entropy:

$$\lim_{n\to\infty} \frac{1}{n} H_{\min}^{\epsilon}(A^n|B^n)_{\rho^{\otimes n}} = H(A|B)_\rho , \tag{16}$$

where $\epsilon > 0$ is an arbitrary smoothing parameter. This is known as the *asymptotic equipartition property* [12]. Thus, in the limit of infinitely many channel uses, where the asymptotic quantity is relevant, the min-entropy reproduces the conditional von Neumann entropy.

To gain some intuition for $H_{\min}(A|E)_\rho$, we now have a look at some special cases. For these special cases, we assume that $\mathcal{H}_{A'} \simeq \mathcal{H}_A \simeq \mathcal{H}_B \simeq \mathcal{H}_E$. Assume that initially, the state $\rho_{AA'}$ is maximally entangled, i.e. $\rho_{AA'} = \Phi_{AA'}$ for some $\Phi_{AA'} \in \Gamma_{AA'}$ analogous to (15). We think of the channel purification $U_{A'E'\to BE}$ as being controlled by Eve.

- If the adversary Eve leaves system A' untouched, i.e. the channel $\Theta_{A'\to B}$ is the identity channel (or any other unitary channel), then $\rho_{AE} = d_A^{-1}\mathbb{1}_A \otimes \rho_E$ for some state ρ_E of system E. In that case, $H_{\min}(A|E)_\rho = \log d_A$, and we say that there is no decoherence.

- In the other extreme case, Eve snatches away the system A' and forwards an uncorrelated system to Bob. In this case, $\rho_{AE} \in \Gamma_{AE}$ with Γ_{AE} analogous to equation (15) (maximal entanglement between A and E). Then, $H_{\min}(A|E)_\rho = -\log d_A$, and we say that we have full decoherence.

- As an intermediate case, we might consider the case where Eve interferes such that she does not end up with maximal entanglement with Alice but such that she is classically correlated with Alice in some basis, i.e. $\rho_{AE} = d_A^{-1}\sum_k |k\rangle\langle k|_A \otimes |k\rangle\langle k|_E$. In that case, $H_{\min}(A|E)_\rho = 0$, and we speak of partial decoherence.

Supplementary Note 2: Decoherence estimation through CHSH tests in quantum theory

Introduction

Our goal is to show that Alice and Bob can estimate the decoherence by performing a Bell experiment. We pose it as a feasibility problem: *is it possible to observe certain statistics in a Bell experiment given a certain level of decoherence*? Solving this problem allows us to determine and plot the *feasible region* in the space of suitably chosen parameters.

We look at the simplest Bell experiment, known as the Clauser-Horne-Shimony-Holt (CHSH) [13] scenario. If ρ_{AB} is the state that Alice and Bob share and A_j, B_k for $j,k \in \{0,1\}$ are the observables they perform, then the CHSH value equals

$$\beta = \mathsf{Tr}\big([A_0 \otimes B_0 + A_0 \otimes B_1 + A_1 \otimes B_0 - A_1 \otimes B_1]\rho_{AB}\big). \tag{17}$$

As explained previously the min-entropy $H_{\min}(A|E)$ defined in Eq. (13) captures the notion of decoherence between Alice and Bob (although note that high min-entropy corresponds to low decoherence and vice versa). Since the range of values that the min-entropy takes depends on the dimension of Alice's system (denoted by d_A), it is only meaningful

to compare scenarios in which d_A is fixed. For simplicity, we consider the simplest non-trivial scenario in which the subsystems held by Alice and Bob are qubits, $d_A = d_B = 2$.

We define the feasible region $\mathcal{S}$ as follows. A pair of real numbers (u, v), where $u \in [-1, 1]$ and $v \in [0, 2\sqrt{2}]$ belongs to $\mathcal{S}$ if there exists a tripartite state ρ_{ABE} and binary observables A_0, A_1 on $\mathcal{H}_A$ and B_0, B_1 on $\mathcal{H}_B$ such that

- subsystems A and B are qubits: $\dim \mathcal{H}_A = \dim \mathcal{H}_B = 2$
- The conditional min-entropy of A given E equals u: $H_{\min}(A|E) = u$.
- The CHSH value given by Eq. (17) equals v: $\beta = v$.

First note that a CHSH value of $v \leqslant 2$ can be achieved using trivial measurements (namely $\{\mathbb{1}, 0\}$) acting on an arbitrary state. Therefore, for $v \leqslant 2$ all values of $u \in [-1, 1]$ are allowed. For the remainder of the argument we implicitly assume that $v > 2$ and the following intuitive argument shows why certain pairs (u, v) must indeed be forbidden. Consider a point $u \approx -1$ and $v > 2$. According to the operational meaning of the min-entropy (14), $u \approx -1$ means that Eve can recover the maximally entangled state with Alice with fidelity close to unity, which clearly allows Alice and Eve to violate the CHSH inequality. On the other hand, since $v > 2$ Alice also observes a CHSH violation with Bob. This violates the monogamy relation for tripartite three-qubit states proved in Ref. [14], which states that Alice can violate the CHSH inequality with at most one party (even if she is allowed to use different measurements for different scenarios). This simple argument leads to the conclusion that the region $u \approx -1$ and $v > 2$ is forbidden. In the remainder of this section we show that the non-trivial part of the feasible region $\mathcal{S}$ can be fully characterized by a single inequality.

Theorem 1: *A pair of real numbers (u, v) where $u \in [-1, 1]$ and $v \in (2, 2\sqrt{2}]$ belongs to the feasible region $\mathcal{S}$ if and only if*

$$u \geqslant f(v), \tag{18}$$

where

$$f(v) := 3 - 2 \log \max_{c_z} \left(2\sqrt{1 + c_z} + \sqrt{1 - c_z + \frac{v}{\sqrt{2}}} + \sqrt{1 - c_z - \frac{v}{\sqrt{2}}} \right), \tag{19}$$

where the maximization is taken over

$$-1 \leqslant c_z \leqslant 1 - \frac{v}{\sqrt{2}}. \tag{20}$$

While the definition of f might seem complicated, it is straightforward to see that f is monotonically increasing in v and evaluating $f(v)$ numerically for a particular value of v is straightforward since the function to be maximized is concave. The feasible region $\mathcal{S}$ is plotted in Supplementary Fig. 4.

The proof of Theorem 1 is conceptually simple, but it requires a wide array of technical tools, which we present in the Preliminaries subsection. In the two subsequent subsections, we prove the direct and converse parts of Theorem 1, respectively.

Preliminaries

Definition 2: *Let $\mathcal{H}_A$, $\mathcal{H}_B$ be Hilbert spaces of dimension d. A* **generalized Bell basis** *for $\mathcal{H}_A \otimes \mathcal{H}_B$ is a set $\{|\Phi_j\rangle\}_{j=1}^{d^2}$ of d^2 pure states on $\mathcal{H}_A \otimes \mathcal{H}_B$ which satisfy*

$$\mathrm{Tr}_A |\Phi_j\rangle\langle\Phi_j| = \frac{\mathbb{1}_B}{d}, \quad \mathrm{Tr}_B |\Phi_j\rangle\langle\Phi_j| = \frac{\mathbb{1}_A}{d} \quad \text{for } j = 1, \ldots, d^2 \text{ and} \tag{21}$$

$$\sum_{j=1}^{d^2} |\Phi_j\rangle\langle\Phi_j| = \mathbb{1}_A \otimes \mathbb{1}_B. \tag{22}$$

A state $\rho_{AB} \in \mathcal{S}(\mathcal{H}_A \otimes \mathcal{H}_B)$ is called **Bell-diagonal** *if it is diagonal in some generalized Bell basis, i.e. if there exists a probability distribution $\{p_j\}_{j=1}^{d^2}$ such that*

$$\rho_{AB} = \sum_{j=1}^{d^2} p_j |\Phi_j\rangle\langle\Phi_j|. \tag{23}$$

Lemma 3: *Let $A \geqslant 0$ be a positive semi-definite operator, let Π_A be the projector on its support and let $|b\rangle$ be a normalized vector. Then $A \geqslant |b\rangle\langle b|$ iff*

$$\Pi_A|b\rangle = |b\rangle \quad \text{and} \quad \langle b|A^{-1}|b\rangle \leqslant 1. \tag{24}$$

Note that since A might not be invertible, A^{-1} is only defined on the support of A.

Two-qubit states

A two-qubit state written in the Pauli basis takes the form

$$\rho_{AB} = \frac{1}{4}\big(\mathbb{1}_A \otimes \mathbb{1}_B + \sum_j a_j\sigma_j \otimes \mathbb{1}_B + \mathbb{1}_A \otimes \sum_j b_j\sigma_j + \sum_{j,k} T_{jk}\sigma_j \otimes \sigma_k\big), \tag{25}$$

where all the summations go over $\{x, y, z\}$. It is known that for every state there exists a local unitary $U_A \otimes U_B$ which diagonalizes the correlation tensor (i.e. ensures that $T_{jk} = 0$ for $j \neq k$) and since all the properties we consider are invariant under local unitaries we can make this assumption without loss of generality. We denote these diagonal entries T_{xx}, T_{yy} and T_{zz} by c_x, c_y and c_z, respectively, which simplifies the expression to

$$\rho_{AB} = \frac{1}{4}\big(\mathbb{1}_A \otimes \mathbb{1}_B + \sum_j a_j\sigma_j \otimes \mathbb{1}_B + \mathbb{1}_A \otimes \sum_j b_j\sigma_j + \sum_j c_j\sigma_j \otimes \sigma_j\big). \tag{26}$$

Without loss of generality, we assume that $|c_x| \geqslant |c_y| \geqslant |c_z|$ and $c_x, c_y \geqslant 0$. As shown in Ref. [15] every Bell-diagonal state of two qubits (up to local unitaries which, again, we can safely ignore) can be written as

$$\rho_{AB} = \sum_{j=1}^{4} p_j|\Phi_j\rangle\langle\Phi_j|, \tag{27}$$

where $\{p_j\}_{j=1}^4$ is a probability distribution and $|\Phi_{1,2}\rangle = \frac{|00\rangle \pm |11\rangle}{\sqrt{2}}$ and $|\Phi_{3,4}\rangle = \frac{|01\rangle \pm |10\rangle}{\sqrt{2}}$. It is easy to verify that

$$\rho_{AB} = \frac{1}{4}\big(\mathbb{1}_A \otimes \mathbb{1}_B + \sum_j c_j\sigma_j \otimes \sigma_j\big), \tag{28}$$

where

$$\begin{aligned} c_x &= p_1 - p_2 + p_3 - p_4\,, \\ c_y &= -p_1 + p_2 + p_3 - p_4\,, \\ c_z &= p_1 + p_2 - p_3 - p_4\,. \end{aligned} \tag{29}$$

Non-locality

Definition 4: *For a bipartite quantum state ρ_{AB} the maximum CHSH value is defined as*

$$\beta_{\max}(\rho_{AB}) := \max_{A_0,A_1,B_0,B_1} \mathsf{Tr}\big[(A_0 \otimes B_0 + A_0 \otimes B_1 + A_1 \otimes B_0 - A_1 \otimes B_1)\rho_{AB}\big], \tag{30}$$

where the maximization is taken over all Hermitian, binary observables.

Note that for all states $\beta_{\max} \geqslant 2$ and we say that the state violates the CHSH inequality if $\beta_{\max} > 2$. It was shown in Ref. [16] that if ρ_{AB} is a state of two qubits then the value of $\beta_{\max}$ is fully determined by the correlation tensor. Adopting the convention $|c_x| \geqslant |c_y| \geqslant |c_z|$ we have

$$\beta_{\max}(\rho_{AB}) = \begin{cases} 2 & \text{if} \quad c_x^2 + c_y^2 \leqslant 1, \\ 2\sqrt{c_x^2 + c_y^2} & \text{otherwise.} \end{cases} \tag{31}$$

To derive a bound on the min-entropy $H_{\min}(A|E)_\rho$, we will use a closely related quantity, namely the *max-entropy.*

Definition 5: *For a bipartite quantum state ρ_{AB} the* ***conditional max-entropy*** *(or just* ***max-entropy****) is defined as*

$$H_{\max}(A|B) = \max_{\sigma_B} \log d_A F^2(\rho_{AB}, \pi_A \otimes \sigma_B)\,, \tag{32}$$

where π_A is the maximally mixed state on A and the maximization is taken over all states on B.

The proof uses the following known properties of the min- and max-entropies.

Lemma 6 (Duality, [11])**:** *Let ρ_{ABC} be a tripartite state. Then*

$$H_{\max}(A|B)_\rho + H_{\min}(A|C)_\rho \geqslant 0,$$

and the equality holds iff ρ_{ABC} is pure.

Lemma 7 (Data-processing inequality, [8])**:** *For an arbitrary tripartite state ρ_{ABC} we have*

$$H_{\max}(A|B) \geqslant H_{\max}(A|BC). \tag{33}$$

Lemma 8 (Conditioning on classical information, Proposition 4.6 of [17])**:** *Let ρ_{ABK} be a tripartite state where K is a classical register:*

$$\rho_{ABK} = \sum_k p_k\, \tau^k_{AB} \otimes |k\rangle\langle k|. \tag{34}$$

Then

$$H_{\max}(A|BK)_{\rho_{ABK}} = \log\Big(\sum_k p_k\, 2^{H_{\max}(A|B)_{\tau^k_{AB}}}\Big). \tag{35}$$

Finally, we need an explicit expression for the max-entropy of a Bell-diagonal state. Note that by assumption $d_A = d_B = d$.

Lemma 9: *Let ρ_{AB} be a Bell-diagonal state of form (23). Then the conditional max-entropy equals*

$$H_{\max}(A|B) = -\log d + 2\log\Big(\sum_j \sqrt{p_j}\Big). \tag{36}$$

To prove Lemma 9 we use the fact that the optimization problem which appears in the definition of the max-entropy (32) can be written as a semidefinite program (SDP) [18]. More specifically, given ρ_{AB} we have $H_{\max}(A|B) = \log\lambda$, where λ is the value of the following SDP for ρ_{ABC} being an arbitrary purification of ρ_{AB}

`PRIMAL` :	minimize	μ	`DUAL` :	maximize	$\mathrm{Tr}(\rho_{ABC} Y_{ABC})$
	subject to	$\mu \mathbb{1}_B \geqslant \mathrm{tr}_A(Z_{AB})$		subject to	$\mathrm{Tr}_C(Y_{ABC}) \leqslant \mathbb{1}_A \otimes \sigma_B$
		$Z_{AB} \otimes \mathbb{1}_C \geqslant \rho_{ABC}$			$\mathrm{Tr}\sigma_B \leqslant 1$
		$Z_{AB} \in \mathcal{P}(\mathcal{H}_{AB})$			$Y_{ABC} \in \mathcal{P}(\mathcal{H}_{ABC})$
		$\mu \geqslant 0$			$\sigma_B \in \mathcal{P}(\mathcal{H}_B)$

where $\mathcal{P}(\mathcal{H})$ denotes the set of positive semi-definite operators acting on $\mathcal{H}$. By providing feasible solutions for the `PRIMAL` and the `DUAL` we show that for Bell-diagonal states

$$\lambda = \frac{1}{d}\Big(\sum_j \sqrt{p_j}\Big)^2 \tag{37}$$

which is precisely the statement of Lemma 9.

Proof. Let $\rho_{ABC} = |\psi_{ABC}\rangle\langle\psi_{ABC}|$ be a purification of ρ_{AB}, e.g.

$$|\psi_{ABC}\rangle = \sum_j \sqrt{p_j}|\Phi_j\rangle \otimes |j\rangle. \tag{38}$$

For the `PRIMAL` consider

$$Z_{AB} = \Big(\sum_j \sqrt{p_j}\Big) \sum_k \sqrt{p_k}|\Phi_k\rangle\langle\Phi_k|, \tag{39}$$

$$\mu = \frac{1}{d}\Big(\sum_j \sqrt{p_j}\Big)^2. \tag{40}$$

Clearly, $Z_{AB} \geqslant 0$, $\mu \geqslant 0$ and since $\mathsf{Tr}_A(Z_{AB}) = \frac{1}{d}\big(\sum_j \sqrt{p_j}\big)^2 \mathbb{1}_B$ the first constraint is easy to check. The last inequality we need to check is

$$\Big(\sum_j \sqrt{p_j}\Big) \sum_k \sqrt{p_k}|\Phi_k\rangle\langle\Phi_k| \otimes \mathbb{1}_C \geqslant \rho_{ABC}. \tag{41}$$

We apply Lemma 3 to $A = Z_{AB} \otimes \mathbb{1}_C$ and $|b\rangle = |\psi_{ABC}\rangle$. The projector on the support of $Z_{AB} \otimes \mathbb{1}_C$ equals

$$\Pi = \sum_{j:p_j>0} |\Phi_j\rangle\langle\Phi_j| \otimes \mathbb{1}_C \tag{42}$$

and it is easy to verify that $\Pi|\psi_{ABC}\rangle = |\psi_{ABC}\rangle$. Moreover, since $(Z_{ABC})^{-1} = (Z_{AB})^{-1} \otimes \mathbb{1}_C$ we have

$$\Big(\sum_m \sqrt{p_m}\langle\Phi_m| \otimes \langle m|\Big)\Bigg(\Big(\sum_j \sqrt{p_j}\Big)^{-1} \sum_{k:p_k>0} \frac{1}{\sqrt{p_k}}|\Phi_k\rangle\langle\Phi_k| \otimes \mathbb{1}_C\Bigg)\Big(\sum_n \sqrt{p_n}|\Phi_n\rangle \otimes |n\rangle\Big) \tag{43}$$

$$= \Big(\sum_j \sqrt{p_j}\Big)^{-1}\Big(\sum_m \sqrt{p_m}\langle\Phi_m| \otimes \langle m|\Big)\Big(\sum_{n:p_n>0} |\Phi_n\rangle \otimes |n\rangle\Big) = 1. \tag{44}$$

Showing that Z_{AB} and μ constitute a valid solution to the `PRIMAL` implies that $\lambda \leqslant \frac{1}{d}\big(\sum_j \sqrt{p_j}\big)^2$. For the `DUAL` consider

$$Y_{ABC} = \frac{1}{d}\sum_{jk} |\Phi_j\rangle\langle\Phi_k| \otimes |j\rangle\langle k|, \tag{45}$$

$$\sigma_B = \frac{\mathbb{1}_B}{d}. \tag{46}$$

Note that Y_{ABC} is proportional to a rank-1 projector. The first constraint gives

$$\mathsf{Tr}_C(Y_{ABC}) = \frac{1}{d}\sum_j |\Phi_j\rangle\langle\Phi_j| = \frac{1}{d}\,\mathbb{1}_A \otimes \mathbb{1}_B = \mathbb{1}_A \otimes \sigma_B \tag{47}$$

and the remaining ones are easily verified to be true. The value of this solution equals $\mathsf{Tr}(\rho_{ABC}Y_{ABC}) = \frac{1}{d}\big(\sum_j \sqrt{p_j}\big)^2$ which implies that $\lambda \geqslant \frac{1}{d}\big(\sum_j \sqrt{p_j}\big)^2$. □

Sufficiency of considering Bell-diagonal states

To prove the converse part of Theorem 1, we will use the following argument, which is similar in spirit and inspired by the symmetrization argument presented in Ref. [19].

Lemma 10: *Let ρ_{AB} be an arbitrary state of two qubits. Then, there exists a Bell-diagonal state σ_{AB} which satisfies*

$$\beta_{\max}(\rho_{AB}) = \beta_{\max}(\sigma_{AB}) \quad \text{and} \quad H_{\max}(A|B)_\sigma \geqslant H_{\max}(A|B)_\rho. \tag{48}$$

Proof. We present an explicit construction of σ_{AB} which meets the requirements. According to Eq. (26), ρ_{AB} can be written as

$$\rho_{AB} = \frac{1}{4}\big(\mathbb{1}_A \otimes \mathbb{1}_B + \sum_j a_j \sigma_j \otimes \mathbb{1}_B + \mathbb{1}_A \otimes \sum_j b_j \sigma_j + \sum_j c_j \sigma_j \otimes \sigma_j\big). \tag{49}$$

Moreover, consider the following random unitary channel

$$\Lambda(\rho_{AB}) = \frac{1}{4}\sum_{j=1}^{4}(U_j \otimes U_j)\rho_{AB}(U_j^\dagger \otimes U_j^\dagger), \tag{50}$$

where $U_1 = \mathbb{1}$, $U_2 = \sigma_x$, $U_3 = \sigma_y$ and $U_4 = \sigma_z$. It is easy to verify that for $j \in \{x, y, z\}$

$$\Lambda(\sigma_j \otimes \mathbb{1}_B) = \Lambda(\mathbb{1}_A \otimes \sigma_j) = 0 \tag{51}$$

because each Pauli operator commutes with identity and itself but anticommutes with the other two unitaries. This implies that $\sigma_{AB} = \Lambda(\rho_{AB})$ is Bell-diagonal. Moreover, one can check that the map preserves the correlation tensor, i.e. for $j \in \{x, y, z\}$

$$\Lambda(\sigma_j \otimes \sigma_j) = \sigma_j \otimes \sigma_j, \tag{52}$$

which implies that $\beta_{\max}(\rho_{AB}) = \beta_{\max}(\sigma_{AB})$. To check the last property consider the following state

$$\sigma_{ABK} = \frac{1}{4}\sum_{j=1}^{4}(U_j \otimes U_j)\rho_{AB}(U_j^\dagger \otimes U_j^\dagger) \otimes |j\rangle\langle j|. \tag{53}$$

By the data processing inequality, we have $H_{\max}(A|B)_\sigma \geqslant H_{\max}(A|BK)_\sigma$ and by conditioning on classical information we have

$$H_{\max}(A|BK)_\sigma = \log\Big(\sum_{j=1}^{4}\frac{1}{4}\cdot 2^{H_{\max}(A|B)_{\tau^j}}\Big), \tag{54}$$

$$\text{where} \quad \tau_{AB}^j = (U_j \otimes U_j)\rho_{AB}(U_j^\dagger \otimes U_j^\dagger). \tag{55}$$

Since the max-entropy is invariant under local unitaries we have $H_{\max}(A|B)_{\tau^j} = H_{\max}(A|B)_\rho$ for $j \in \{x, y, z\}$ which implies that

$$H_{\max}(A|B)_\sigma \geqslant H_{\max}(A|BK)_\sigma = H_{\max}(A|B)_\rho. \tag{56}$$

□

The final technical lemma concerns the problem of maximizing the max-entropy of a Bell-diagonal state of two qubits whose maximal CHSH violation is fixed.

Lemma 11: *Let ρ_{AB} be a Bell-diagonal state of two qubits, whose maximal CHSH violation equals $\beta \in (2, 2\sqrt{2}]$. Then, the max-entropy of ρ_{AB} satisfies the following inequality*

$$H_{\max}(A|B) \leqslant -f(\beta) \tag{57}$$

for function f defined in Eq. (19). Moreover, there exists a state which saturates this inequality.

Proof. According to Lemma 9 the max-entropy of a Bell-diagonal state of two qubits equals

$$H_{\max}(A|B) = -1 + 2\log\Big(\sum_{j=1}^{4}\sqrt{p_j}\Big). \tag{58}$$

Here, it is convenient to express the probabilities through the correlation coefficients c_x, c_y, c_z. Inverting Eqs. (29) gives

$$p_1 = \frac{1}{4}(1 + c_x - c_y + c_z), \quad p_2 = \frac{1}{4}(1 - c_x + c_y + c_z), \tag{59}$$

$$p_3 = \frac{1}{4}(1 + c_x + c_y - c_z), \quad p_4 = \frac{1}{4}(1 - c_x - c_y - c_z), \tag{60}$$

which allows us to write

$$H_{\max}(A|B) = -3 + 2\log g(c_x, c_y, c_z), \tag{61}$$

where

$$g(c_x, c_y, c_z) = \sqrt{1 + c_x - c_y + c_z} + \sqrt{1 - c_x + c_y + c_z} + \sqrt{1 + c_x + c_y - c_z} + \sqrt{1 - c_x - c_y - c_z}. \tag{62}$$

In the space of correlation coefficients the feasible set are the triples (c_x, c_y, c_z) for which the function $g(c_x, c_y, c_z)$ is well-defined (the expressions under the roots must be non-negative). As before, we assume without loss of generality that $|c_x| \geqslant |c_y| \geqslant |c_z|$ and $c_x, c_y \geqslant 0$. Then, the maximal CHSH violation (we are only interested in states that violate the CHSH inequality) is given by Eq. (31)

$$\beta = 2\sqrt{c_x^2 + c_y^2}.$$

Since in our case β is fixed, the angular parametrization takes the form

$$c_x = \frac{q}{\sqrt{2}} \sin\Big(\phi + \frac{\pi}{4}\Big) \quad \text{and} \quad c_y = \frac{q}{\sqrt{2}} \cos\Big(\phi + \frac{\pi}{4}\Big),$$

where $q = \frac{\beta}{\sqrt{2}}$ and $\phi \in [0, \pi/4]$ (which ensures $c_x \geqslant c_y \geqslant 0$). Note that

$$c_x + c_y = q\cos\phi,$$
$$c_x - c_y = q\sin\phi.$$

It is easy to check that the allowed range of c_z is

$$q\sin\phi - 1 \leqslant c_z \leqslant 1 - q\cos\phi.$$

Note that we should also impose the condition $|c_z| \leqslant |c_y|$ but as it turns out the optimal solution will satisfy it even if we do not include it explicitly. To maximize the max-entropy it is sufficient to maximize function g defined in Eq. (62), which in the angular parametrization equals

$$g(\phi, c_z) = \sqrt{1 + c_z + q\sin\phi} + \sqrt{1 + c_z - q\sin\phi} + \sqrt{1 - c_z + q\cos\phi} + \sqrt{1 - c_z - q\cos\phi}, \tag{63}$$

over

$$\mathcal{R} = \big\{(\phi, c_z) : \phi \in [0, \pi/4],\ q\sin\phi - 1 \leqslant c_z \leqslant 1 - q\cos\phi\big\}. \tag{64}$$

The maximum is achieved either in the interior (denoted by $\mathcal{R}_{\text{int}}$) or at the boundary. Let us start by ruling out the first option. Function g is differentiable everywhere in $\mathcal{R}_{\text{int}}$ and the partial derivatives are

$$\frac{\partial g}{\partial c_z} = \frac{1}{2\sqrt{1 + c_z + q\sin\phi}} + \frac{1}{2\sqrt{1 + c_z - q\sin\phi}} + \frac{-1}{2\sqrt{1 - c_z + q\cos\phi}} + \frac{-1}{2\sqrt{1 - c_z - q\cos\phi}}, \tag{65}$$

$$\frac{\partial g}{\partial \phi} = \frac{q\cos\phi}{2\sqrt{1 + c_z + q\sin\phi}} + \frac{-q\cos\phi}{2\sqrt{1 + c_z - q\sin\phi}} + \frac{-q\sin\phi}{2\sqrt{1 - c_z + q\cos\phi}} + \frac{q\sin\phi}{2\sqrt{1 - c_z - q\cos\phi}}. \tag{66}$$

To prove that there is no maximum in the interior, it suffices to show that there is no $(\phi, c_z) \in \mathcal{R}_{\text{int}}$ such that both derivatives vanish $\frac{\partial g}{\partial c_z} = \frac{\partial g}{\partial \phi} = 0$. To do this we consider the following linear combination

$$s(\phi, c_z) = 2\sin\phi \cdot \frac{\partial g}{\partial c_z} + \frac{2}{q} \cdot \frac{\partial g}{\partial \phi} = \frac{\sin\phi + \cos\phi}{\sqrt{1 + c_z + q\sin\phi}} + \frac{\sin\phi - \cos\phi}{\sqrt{1 + c_z - q\sin\phi}} + \frac{-2\sin\phi}{\sqrt{1 - c_z + q\cos\phi}}$$

and show that $s(\phi, c_z) = 0$ has no solution in $\mathcal{R}_{\text{int}}$. Since the last term of $s(\phi, c_z)$ is negative, a necessary condition for $s(\phi, c_z) = 0$ is that the sum of the first two terms is non-negative, which is equivalent to

$$\frac{\sin\phi + \cos\phi}{\sqrt{1 + c_z + q\sin\phi}} \geqslant \frac{\cos\phi - \sin\phi}{\sqrt{1 + c_z - q\sin\phi}}.$$

This can be rearranged to give

$$c_z \geqslant \frac{q}{2\cos\phi} - 1,$$

which contradicts the second inequality in the definition of $\mathcal{R}_{\text{int}}$ as shown below.

$$c_z \geqslant \frac{q}{2\cos\phi} - 1 \quad \text{and} \quad 1 - q\cos\phi > c_z \tag{67}$$

$$\implies 1 - q\cos\phi > \frac{q}{2\cos\phi} - 1 \iff \frac{1}{2\cos\phi} + \cos\phi < \frac{2}{q}. \tag{68}$$

It is easy to check that the left-hand side of the final inequality is always at least $\sqrt{2}$, while the right hand side is always at most $\sqrt{2}$. This proves that the final (strict) inequality is always false, which implies that $s(\phi, c_z) = 0$ has no solutions in $\mathcal{R}_{\text{int}}$ and that $g(\phi, c_z)$ has no maximum in $\mathcal{R}_{\text{int}}$.

The boundaries $c_z = q\sin\phi - 1$ and $c_z = 1 - q\cos\phi$ correspond to one of the expression under the roots being zero. Since the square root function has infinite slope at 0, such solutions cannot be optimal. Therefore, the maximum must be achieved at the boundary $\phi = 0$. Combining Equations (61) and (63) and setting $\phi = 0$ leads directly to the statement of the lemma.

To show that the solution of the optimization problem satisfies $|c_z| \leqslant |c_y|$, it is sufficient to show that for $\phi = 0$ and $c_z = -c_y = -q/2$ the partial derivative $\partial g/\partial c_z$ is strictly positive. □

The direct part

Here, we show (by an explicit construction) that points described by $v \in (2, 2\sqrt{2}]$ and $f(v) \leqslant u \leqslant 1$ are allowed. Lemma 11 shows that for $v \in (2, 2\sqrt{2}]$ there exists a Bell-diagonal state of two qubits whose max-entropy equals

$$H_{\max}(A|B) = -f(v). \tag{69}$$

By duality (Lemma 6), if ρ_{ABE} is an arbitrary purification, the conditional min-entropy equals

$$H_{\min}(A|E) = f(v). \tag{70}$$

In this example $u = f(v)$, which corresponds to a point lying precisely on the boundary defined in Theorem 1. In order to obtain higher values of u (all the way up to 1), it suffices to apply noise of appropriate strength to subsystem E.

The converse part

Here, we show that every feasible point (u, v) must satisfy $u \geqslant f(v)$. Consider a state ρ_{ABE} for which $H_{\min}(A|E)_\rho = u$ and which for some measurements achieves the CHSH value of v. Clearly, $\beta_{\max}(\rho_{AB}) \geqslant v$ and by Lemma 6 $H_{\max}(A|B)_\rho \geqslant -u$. Applying the symmetrization argument (Lemma 10) gives rise to a Bell-diagonal state σ_{AB} such that $H_{\max}(A|B)_\sigma \geqslant -u$ and $\beta_{\max}(\sigma_{AB}) \geqslant v$. By Lemma 11 these quantities must satisfy

$$H_{\max}(A|B)_\sigma \leqslant -f\big(\beta_{\max}(\sigma_{AB})\big), \tag{71}$$

which implies that

$$u \geqslant -H_{\max}(A|B)_\sigma \geqslant f\big(\beta_{\max}(\sigma_{AB})\big) \geqslant f(v), \tag{72}$$

where the last inequality follows from the fact that f is monotonically increasing.

Supplementary Note 3: Decoherence estimation through CHSH tests in GPTs

In this appendix, we are going to develop a framework for decoherence analysis in analogy to Supplementary Note 2, but without assuming that nature is correctly described by quantum theory. Instead, we will work in a framework that makes only minimal assumptions about the probabilistic structure of measurements. This allows to make statements in cases where quantum theory might not be a correct description of nature.

In the first subsection, we define a framework for probabilistic theories that has become a standard one in the literature. Besides defining the core structure, we explain how we extend this framework to make it suitable for analyzing tripartite states, in a way that allows us to make a decoherence analysis that is analogous to the quantum case.

In the second subsection, we will define a decoherence quantity $\mathrm{Dec}(A|E)_\omega$ for GPTs as an analogue of the quantum min-entropy $H_{\min}(A|E)_\rho$. This will be our quantity of interest for the decoherence analysis for GPTs. We will first motivate an expression for $\mathrm{Dec}(A|E)_\omega$, inspired by expression (14) for the min-entropy in the quantum case. This expression will require us to say what a maximally entangled state in a GPT is.

The third subsection is devoted to finding a bound on our decoherence quantity in terms of the CHSH winning probability for Alice and Bob. This is a measurable quantity in the case where the channel is an *iid* (for *independent and identically distributed*) channel, meaning that it behaves identically in repeated uses of the channel without building up correlations amongst systems going through the channel in different uses of it. This is a practically relevant case, giving our bound a practical meaning. This bound allows us to infer non-trivial statements about decoherence from measured data when, apart from the iid assumption, we assume only very little about the behavior of nature. We approach our bound by first bounding our fidelity-based decoherence quantity by a trace distance-based quantity. We will then bound this trace distance-based quantity in terms of the CHSH winning probability for Alice and Bob by a quantity that can be expressed as a linear program.

Finally, in the fourth subsection, we show how our bound can be expressed as a linear program and present the numerical results. This is followed by a discussion of the physical interpretation of our numerical findings.

The framework

A basic framework for GPTs

Frameworks for probabilistic theories in which quantum theory and classical theory can be formulated as special cases have already been considered some decades ago [20–22]. After some period of oblivion, a seminal paper by Hardy [23] caused a revival in the interest in such frameworks (see, for example, [24–29] and references therein). Today, they are generally referred to as frameworks for *generalized probabilistic theories* [30].

We formalize our decoherence analysis for GPTs in the *abstract state space* framework [31–34]. It is one rigorous formalization of what a generalized probabilistic theory is, amongst a few equivalent or closely related ones that can be found in the literature (see the references cited above). We prefer it for its concise and precise formulation. For the sake of brevity, we will not go far beyond the mere mathematical definitions related to abstract state spaces here. For a detailed introduction to abstract state spaces, see [35].

Definition 12: *An* ***abstract state space*** *is a triple (V, V^+, u), where V is a finite- dimensional real vector space, V^+ is a cone[2] in V which is closed[3] and generating[4] and $u \in V^*$ is a linear functional[5] on V such that $u(\omega) > 0$ for all $\omega \in V^+ \setminus \{0\}$. The functional u is called the* ***unit effect****.*

Definition 13: *For an abstract state space (V, V^+, u), we define the following induced structure (see Supplementary Fig. 5):*
The ***normalized states*** *are the elements of the set*

$$\Omega := \{\omega \in V^+ \mid u(\omega) = 1\}\,. \tag{73}$$

The ***subnormalized states*** *are the elements of the set*

$$\Omega^{\leqslant} := \{\omega \in V^+ \mid u(\omega) \leqslant 1\}\,. \tag{74}$$

The ***effects*** *are the elements of the set*

$$\mathcal{E} := \{e \in V^* \mid 0 \leqslant e(\omega) \leqslant 1 \ \forall \omega \in \Omega\}\,. \tag{75}$$

---

[2] A subset $V^+ \subseteq V$ is a *cone* in V if
(C1) $V^+ + V^+ \subseteq V^+$,
(C2) $\alpha V^+ \subseteq V^+$ for all $\alpha \geqslant 0$,
(C3) $V^+ \cap (-V^+) = \{0\}$,

[3] We assume the standard topology on V, i.e. the only linear Hausdorff topology on V.
[4] A cone $V^+ \subseteq V$ is *generating* if $V^+ - V^+ = V$.
[5] For a finite-dimensional vector space V, we denote by V^* the dual space of V, i.e. the vector space of linear functionals on V.

*The **measurements** are the elements of the set*

$$\mathcal{M} := \left\{ M \subseteq \mathcal{E} \text{ finite} \;\middle|\; \sum_{e \in M} e = u \right\} . \tag{76}$$

An effect represents a measurement outcome. If a system in a state ω is measured with respect to a measurement $M = \{e_1, \ldots, e_n\}$, then $e_k(\omega)$ is the probability that the measurement yields the outcome associated with e_k.

Example 14 (Quantum theory)**:** *The probabilistic structure of measurements on a (finite-dimensional) quantum system can be formulated as an abstract state space. For a quantum system with an associated Hilbert space $\mathcal{H}$, consider the abstract state space $(V, V^+, u) = (\mathrm{Herm}(\mathcal{H}), \mathrm{Pos}(\mathcal{H}), \mathsf{Tr})$, where $V = \mathrm{Herm}(\mathcal{H})$ is the real vector space of Hermitian operators on $\mathcal{H}$, $V^+ = \mathrm{Pos}(\mathcal{H})$ is the cone of positive operators on $\mathcal{H}$ and $u = \mathsf{Tr}$ is the trace on $\mathcal{H}$. According to Definition 13, this yields the states $\Omega = \{\rho \in \mathrm{Pos}(\mathcal{H}) \mid \mathsf{Tr}(\rho) = 1\}$, which are precisely the density operators on $\mathcal{H}$. Analogously, $\Omega^{\leqslant}$ are the subnormalized density operators. The effects are the functionals induced by POVM elements via the trace, $\mathcal{E} = \{\mathsf{Tr}(P\ \cdot\ ) \mid P \in \mathrm{Pos}(\mathcal{H}), P \leqslant \mathbb{1}_{\mathcal{H}}\}$. Accordingly, the measurements are the sets of functionals that are induced by POVMs, $\mathcal{M} = \{\{\mathsf{Tr}(P_k\ \cdot\ ) \mid P_k \in \{P_k\}_k\} \mid \{P_k\}_k \text{ is a POVM}\}$. This precisely reproduces the structure of measurement statistics in quantum theory. For further details, see [35].* ■

By our definition, $\mathcal{E}$ is the set of *all* linear functionals e such that $0 \leqslant e(\omega) \leqslant 1$ for all $\omega \in \Omega$. The underlying assumption that every such linear functional represents a physical measurement outcome has been called the *no-restriction hypothesis* [28]. A priori, there seems to be no immediate physical reason for this assumption, and some authors have argued about how to weaken this assumption [36]. For our purposes here, it is not relevant whether the no-restriction hypothesis holds, and weakening the assumption complicates the definitions. Thus, we assume it for simplicity.

In the second subsection, we will define a decoherence quantity $\mathrm{Dec}(A|E)_\omega$ analogous to the quantum min-entropy $H_{\min}(A|E)_\rho$. We will take our inspiration from expression (14) for the quantum min-entropy, which involves the fidelity as a measure of closeness of quantum states. Therefore, it is desirable to have a generalization of the fidelity to states in abstract state spaces. Such a generalization is easily found once it is noticed that the quantum fidelity of two states can be expressed as the *Bhattacharyya coefficient* (or *classical fidelity*) of the two probability distributions that the two states induce, minimized over all measurements. More precisely, the quantum fidelity satisfies [37]

$$F(\rho, \sigma) = \min_{\{P_k\}_k} \sum_k \sqrt{\mathsf{Tr}(P_k \rho) \mathsf{Tr}(P_k \sigma)} , \tag{77}$$

where the minimization runs over all POVMs $\{P_k\}_k$ on the Hilbert space on which ρ and σ are defined. The sum in (77) is precisely the Bhattacharyya coefficient of the probability distributions that the POVM $\{P_k\}_k$ induces on the states ρ and σ. This motivates us to define the fidelity for abstract state spaces as follows.

Definition 15: *Let (V, V^+, u) be an abstract state space with normalized states Ω and measurements $\mathcal{M}$. For states $\omega, \tau \in \Omega$, we define the **fidelity** of ω and τ as*

$$F(\omega, \tau) := \inf_{M \in \mathcal{M}} b(\omega, \tau | M) , \quad \text{where} \quad b(\omega, \tau | M) = \sum_{e \in M} \sqrt{e(\omega)} \sqrt{e(\tau)} . \tag{78}$$

*The quantity $b(\omega, \tau | M)$ is the **Bhattacharyya coefficient** (or sometimes called the classical fidelity) of the probability distributions that the measurement M induces on the states ω and τ.*

The fidelity as defined in Definition 15 precisely reduces to the quantum fidelity in the case where the abstract state space is a quantum state space. In addition to the fidelity, in third subsection we will also consider a generalization of the quantum trace distance $D(\rho, \sigma) = \frac{1}{2}\mathsf{Tr}|\rho - \sigma|$ in order to formulate a bound on $\mathrm{Dec}(A|E)_\omega$. Somewhat analogously to the fidelity, the quantum trace distance is equal to the *total variation distance* (or *classical trace distance*) between the two probability distributions that the two states induce, maximized over all measurements [37]:

$$D(\rho, \sigma) = \max_{\{P_k\}_k} \frac{1}{2} \sum_k |\mathsf{Tr}(P_k \rho) - \mathsf{Tr}(P_k \sigma)| . \tag{79}$$

This motivates the following definition.

Definition 16: *Let (V, V^+, u) be an abstract state space with normalized states Ω and measurements $\mathcal{M}$. For states $\omega, \tau \in \Omega$, the* ***trace distance*** *between ω and τ is given by*

$$D(\omega, \tau) := \sup_{M \in \mathcal{M}} d(\omega, \tau | M), \quad \text{where} \quad d(\omega, \tau|M) = \frac{1}{2} \sum_{e \in M} |e(\omega) - e(\tau)| \tag{80}$$

The quantity $d(\omega, \tau|M)$ is the ***total variation distance*** *(or sometimes called the classical trace distance) between the probability distributions that the measurement M induces on the states ω and τ.*

Note that the fidelity and the trace distance take values between 0 and 1 for all states. For squares of the quantities F, b, D and d, we will write the square sign right after the letter, e.g. we will write $F^2(\omega, \tau)$ instead of $(F(\omega, \tau))^2$.

A tripartite framework for GPTs

In the second subsection, we will consider a tripartite situation for the decoherence analysis, analogous to Supplementary Note 1. This requires us to model a tripartite scenario mathematically since such a structure is not induced by an abstract state space (V, V^+, u) alone. We need to specify it as additional structure. Our goal here is to do this with the weakest possible assumptions, resulting in a very general validity of the bounds we derive.

Instead of assuming individual state spaces for every party, we only consider their overall combined state space, modeled by an abstract state space (V, V^+, u) and all its induced structure as in Definitions 12 and 13. This has the advantage that we do not have to make assumptions about how individual state spaces combine to multipartite state spaces, keeping our assumptions weak. For our purposes, the only structure that we need to add to an abstract state space (V, V^+, u) to make it suitable for the description of a tripartite scenario are the local transformations that each individual party can perform. The local measurements of the three parties are then induced by these local transformations.

We consider three parties, which we call Alice (A), Bob (B) and Eve (E) as before. We begin our considerations by assuming that there are three sets $\mathcal{T}_A$, $\mathcal{T}_B$ and $\mathcal{T}_E$, containing all the transformations that Alice, Bob and Eve can perform, respectively. By a transformation, we mean a linear map $T : V \to V$ which maps states to subnormalized states, i.e. $T(\Omega) \subseteq \Omega^{\leqslant}$ (or, equivalently, $T(V^+) \subseteq V^+$ and $(u \circ T)(\omega) \leqslant u(\omega)$ for all $\omega \in V^+$). We can consider the case where several transformations are applied because compositions of transformations are transformations again: If T, T' are linear maps $V \to V$ which map Ω inside $\Omega^{\leqslant}$, then the same is true for the composition $T \circ T'$ (we denote the composition of maps by a $\circ$ symbol).

We assume that the three parties act individually at spatially separated locations. Relativistic considerations lead to the consistency requirement that transformations performed by different parties must commute, e.g. if Alice performs a transformation $T_A \in \mathcal{T}_A$ and Bob performs a transformation $T_B \in \mathcal{T}_B$, then the total transformation must satisfy $T_A \circ T_B = T_B \circ T_A$.

For our purposes, we do not need to specify the sets $\mathcal{T}_A$, $\mathcal{T}_B$ and $\mathcal{T}_E$ any further; the only requirement is that transformations of distinct parties commute. The sets $\mathcal{T}_A$, $\mathcal{T}_B$ and $\mathcal{T}_E$ *define* the systems A, B and E, i.e. we define the individual parties via the transformations that they can perform. This leads us to the following definition.

Definition 17: *A* ***tripartite scenario*** *is a quadruplet*

$$S_{ABE} = ((V, V^+, u), \mathcal{T}_A, \mathcal{T}_B, \mathcal{T}_E), \tag{81}$$

where (V, V^+, u) is an abstract state space, and where

$$\mathcal{T}_A, \mathcal{T}_B, \mathcal{T}_E \subseteq \{T : V \to V \text{ linear} \mid T(\Omega) \subseteq \Omega^{\leqslant}\} \tag{82}$$

are such that for all $P, P' \in \{A, B, E\}$ with $P \neq P'$, it holds that $T_P \circ T_{P'} = T_{P'} \circ T_P$ for all $T_P \in \mathcal{T}_P$ and for all $T_{P'} \in \mathcal{T}_{P'}$. We call the elements of $\mathcal{T}_A$, $\mathcal{T}_B$ and $\mathcal{T}_E$ the ***local transformations*** *of A, B and E, respectively.*

It is absolutely natural to define tripartite scenarios via commuting transformations rather than via a tensor product structure. In quantum theory, the two approaches are equivalent in finite dimensions (we will talk about this below). In more general infinite-dimensional cases, where it is not known whether the two approaches are equivalent, things are usually formalized in a commutative way rather than via tensor products (see [38], for example). Knowing about the equivalence in finite dimensions, we will formulate some quantum examples in the tensor product structure below.

Example 18 (A tripartite quantum scenario)**:** *One can formulate a tripartite situation in quantum theory as a* tripartite scenario*. Based on Example 14, consider the tripartite scenario*

$$
\begin{aligned}
&((\mathrm{Herm}(\mathcal{H}), \mathrm{Pos}(\mathcal{H}), \mathsf{Tr}), \mathcal{T}_A, \mathcal{T}_B, \mathcal{T}_E)\,, \quad \textit{where} && (83)\\
&\mathcal{H} = \mathcal{H}_A \otimes \mathcal{H}_B \otimes \mathcal{H}_E\,, && (84)\\
&\mathcal{T}_A = \{\mathcal{R}_A \otimes \mathbb{1}_B \otimes \mathbb{1}_E \mid \mathcal{R}_A \textit{ is a trace non-increasing CPM on } \mathrm{Herm}(\mathcal{H}_A)\}\,, && (85)\\
&\mathcal{T}_B = \{\mathbb{1}_A \otimes \mathcal{R}_B \otimes \mathbb{1}_E \mid \mathcal{R}_B \textit{ is a trace non-increasing CPM on } \mathrm{Herm}(\mathcal{H}_B)\}\,, && (86)\\
&\mathcal{T}_E = \{\mathbb{1}_A \otimes \mathbb{1}_B \otimes \mathcal{R}_E \mid \mathcal{R}_E \textit{ is a trace non-increasing CPM on } \mathrm{Herm}(\mathcal{H}_E)\}\,, && (87)
\end{aligned}
$$

where CPM stands for completely positive map*. Having tensor product form, the local transformations of different parties commute.* ■

For our purposes, Definition 17 is all the structure one needs to specify. The local measurements are induced by the local transformations. We formalize this via the notion of a local *instrument* [22]. To get an intuition for what an instrument is, consider a Stern-Gerlach experiment. A spin-1/2 particle enters a magnet and undergoes one of two transformations: It either gets deflected upwards or downwards. Which of the two transformations it undergoes is determined probabilistically. Then it hits a screen, which reveals which of the two transformations the particle has undergone. This way, a measurement has been performed in two stages: a probabilistic application of a transformation and a detection. The sum of the probabilities of detecting the particle at the top or the bottom of the screen is one. If the state of the particle is described by a state $\omega \in \Omega$ of an abstract state space, we may model this by a set of two transformations $\{T_{\mathrm{up}}, T_{\mathrm{down}}\}$. Such a set is an instrument. The norm $u(T_{\mathrm{up}}(\omega))$ is the probability that the particle is deflected upwards, and likewise for $u(T_{\mathrm{down}}(\omega))$. Thus, u can be seen to play the role of the screen, detecting the particle. The requirement that the particle must undergo one of the two deflections reads $u \circ T_{\mathrm{up}} + u \circ T_{\mathrm{down}} = u$. The transformation T_{up} is the analogue of the transformation $\rho \mapsto P_{\mathrm{up}} \rho P_{\mathrm{up}}$ in quantum theory, where P_{up} is the projector onto the spin-up state. Since u is given by the trace in quantum theory, the probability for the upward-deflection to occur is given by $\mathsf{Tr}(P_{\mathrm{up}} \rho P_{\mathrm{up}}) = \mathsf{Tr}(P_{\mathrm{up}} \rho)$, which is precisely the Born rule.

A *local* instrument is such a set of transformations where all the transformations are the *local* transformations of one party. This motivates the following definition.

Definition 19 (Local instruments)**:** *For a tripartite scenario $S_{ABE} = ((V, V^+, u), \mathcal{T}_A, \mathcal{T}_B, \mathcal{T}_E)$ with Ω as defined in Definition 13, we define the* ***local instruments*** *as the elements of*

$$
\mathcal{I}_P := \left\{ I_P \subseteq \mathcal{T}_P \ \textit{finite} \ \middle| \ \sum_{T_P \in I_P} u \circ T_P = u \right\} \quad \textit{for } P \in \{A, B, E\}\,. \tag{88}
$$

Example 20 (Local instruments in a tripartite quantum scenario)**:** *Considering the tripartite scenario of Example 18, we get that the local instruments are given by*

$$
\mathcal{I}_P = \left\{ I_P \subseteq \mathcal{T}_P \ \middle| \ \sum_{T_P \in I_P} T_P \textit{ is a TPCPM} \right\} \quad \textit{for } P \in \{A, B, E\}\,.
$$
■

Remark 21 (Local measurements)**:** *The definition of local instruments gives us a notion of local measurements as well. Consider a tripartite scenario $S_{ABE} = ((V, V^+, u), \mathcal{T}_A, \mathcal{T}_B, \mathcal{T}_E)$ with its set of measurements $\mathcal{M}$. It is easily verified that for a local transformation $T_A \in \mathcal{T}_A$, the map $u \circ T_A$ is an effect (as defined in Definition 13). Likewise, for a local instrument $I_A \in \mathcal{I}_A$, the set $\{u \circ T_A \mid T_A \in I_A\}$ is a measurement. We interpret it as a measurement performed by Alice. We can also consider composite measurements where several parties locally perform measurements. For local instruments $I_A \in \mathcal{I}_A$ and $I_B \in \mathcal{I}_B$, for example, the set $\{u \circ T_A \circ T_B \mid T_A \in I_A, T_B \in I_B\}$ is a measurement. We interpret it as a composite measurement where Alice and Bob each perform local measurements, described by I_A and I_B. The analogous holds for other parties and combinations thereof.*

Example 22 (Local measurements in a tripartite quantum scenario)**:** *Based on Examples 18 and 20, we can say how local measurements look like in a tripartite quantum scenario. A local effect of Alice is of the form*

$$
\rho_{ABE} \mapsto \mathsf{Tr}(\mathcal{R}_A \otimes \mathbb{1}_B \otimes \mathbb{1}_E(\rho_{ABE})) \tag{89}
$$

for a trace non-increasing CPM $\mathcal{R}_A$ on $\mathrm{Herm}(\mathcal{H}_A)$*. However, for every such CPM, there is a POVM element P_A on*

$\mathcal{H}_A$ such that[6]

$$\mathrm{Tr}((P_A \otimes \mathbb{1}_B \otimes \mathbb{1}_E)\rho_{ABE}) = \mathrm{Tr}(\mathcal{R}_A \otimes \mathbb{1}_B \otimes \mathbb{1}_E(\rho_{ABE}))\,. \tag{90}$$

This recovers the Born rule. Analogously, a composite measurement where Alice and Bob each perform local measurements consists of local effects of the form

$$\rho_{ABE} \mapsto \mathrm{Tr}(\mathcal{R}_A \otimes \mathcal{R}_B \otimes \mathbb{1}_E(\rho_{ABE})) = \mathrm{Tr}((P_A \otimes P_B \otimes \mathbb{1}_E)\rho_{ABE}) \tag{91}$$

for POVM elements P_A, P_B on $\mathcal{H}_A$, $\mathcal{H}_B$. Thus, in our tripartite quantum example, local measurements reduce to POVM measurements of product form. ■

In Examples 18, 20 and 22, instead of choosing a tensor factorization for $\mathcal{H}$ and setting the local transformations to be acting non-trivially on one tensor factor, we could have chosen sets of transformations that merely commute, without a tensor product structure. The question of whether the resulting measurement statistics in that case would be different from the case with the tensor factor structure is known as *Tsirelson's problem* [39, 40]. More precisely, the question is the following. Let $\mathcal{H}$ be a Hilbert space, let ρ be a density operator on $\mathcal{H}$, let $\{P_k\}_k$, $\{Q_l\}_l$ be POVMs on $\mathcal{H}$ such that $P_k Q_l = Q_l P_k$ for all k, l. Tsirelson's problem is: Does there necessarily exist Hilbert spaces $\mathcal{H}_A$, $\mathcal{H}_B$, a density operator σ on $\mathcal{H}_A \otimes \mathcal{H}_B$ and POVMs $\{R_k\}_k$ on $\mathcal{H}_A$ and $\{S_l\}_l$ on $\mathcal{H}_B$ such that $\mathrm{Tr}(P_k Q_l \rho) = \mathrm{Tr}((R_k \otimes S_l)\sigma)$ for all k, l? In the case where $\mathcal{H}$ is finite-dimensional, the answer is known to be affirmative. For infinite-dimensional Hilbert spaces, the answer is still unknown.

Thus, for finite-dimensional quantum systems, we can restrict ourselves to the case with the tensor product structure without loss of generality. For abstract state spaces, however, an analogous restriction might cause a loss of generality. The advantage of our weak definition of a tripartite scenario is that we do not need to know the answer to an equivalent of Tsirelson's problem for generalized probabilistic theories. The downside is that it makes defining an equivalent of the min-entropy more difficult. We will deal with this issue in the next subsection.

Notation: *From now on, whenever we speak of a tripartite scenario S_{ABE}, we implicitly assume that all its parts and induced structures are denoted as in Definitions 12, 13, 17 and 19 without restating it, i.e. instead of writing "Let $S_{ABE} = ((V, V^+, u), \mathcal{T}_A, \mathcal{T}_B, \mathcal{T}_E)$ be a tripartite scenario, let Ω be its set of normalized states, ...", we will only write "Let S_{ABE} be a tripartite scenario".*

A decoherence quantity for GPTs

Motivation of an expression that quantifies decoherence

We are now going to motivate an expression for the central quantity $\mathrm{Dec}(A|E)_\omega$ for our decoherence analysis for GPTs. We take our inspiration from expression (14) for the quantum min-entropy, which we repeat here for the reader's convenience:

$$H_{\min}(A|E)_\rho = -\log d_A \max_{\mathcal{R}_{E\to A'}} F^2(\Phi_{AA'}, \mathbb{1}_A \otimes \mathcal{R}_{E\to A'}(\rho_{AE}))\,. \tag{14 revisited}$$

There are two issues that prevent us from directly translating expression (14) into our framework. The first issue is that in in the first subsection, to keep our framework as general as possible, we have defined a tripartite scenario with an overall state space (V, V^+, u) with tripartite states Ω. We do not have notions of individual state spaces at hand. Thus, we do not have an analogue of a reduced state ρ_{AE} or of a transformation $\mathcal{R}_{E\to A'}$ from one state space to another.

The second issue is that we do not know what the analogue of a maximally entangled state $\Phi_{AA'}$ in our framework is. We resolve the first issue in the following paragraphs, arriving at an expression for $\mathrm{Dec}(A|E)_\omega$. Further below, we will then define what a maximally entangled state is in our framework.

Expression (14), which involves the state ρ_{AE} and TPCPMs $\mathcal{R}_{E\to A'}$, can be transformed to an expression in which both the state and the TPCPMs are purified (see Supplementary Fig. 6). This expression will be our motivation for the expression for $\mathrm{Dec}(A|E)_\omega$. The maximization over TPCPMs from E to A' is replaced by a maximization over

---

[6] This can be seen from the Kraus representation of $\mathcal{R}_A$: $\mathrm{Tr}(\mathcal{R}_A(\rho_A)) = \mathrm{Tr}(\sum_k F_k \rho_A F_k^\dagger) = \mathrm{Tr}(\sum_k F_k^\dagger F_k \rho_A) = \mathrm{Tr}(P_A \rho_A)$ for $P_A = \sum_k F_k^\dagger F_k$. (We omitted the other tensor factors for brevity.)

unitaries from EE'' to $A'A''$, where E'' and A'' are ancilla systems extending system E and A', respectively. This is precisely the purification (or Stinespring dilation) of a channel as in Supplementary Note 1. Since systems EE'' and $A'A''$ have the same dimension, we can identify their Hilbert spaces and regard the resulting Hilbert space as the Hilbert space of a system E_{tot}. This system involves all subsystems that the third party needs to control in order to bring itself as close as possible to maximal entanglement with Alice. Since $U_{E_{\text{tot}}}$ is a transformation on system E_{tot} alone, we can translate it into our generalized framework.

The state ρ_{AE} is replaced by a purification ρ_{ABE}. We choose the purifying system B to be the channel's output system, which gives us the overall picture of our decoherence analysis as shown in Supplementary Fig. 7.

The following gives a precise formulation of the purification of expression (14). It can be proved using purification and Stinespring dilation. *Let $\mathcal{H}_A$, $\mathcal{H}_E$ be finite-dimensional Hilbert spaces of dimensions d_A, d_E, respectively, let $\rho_{AE} \in \mathcal{S}(\mathcal{H}_A \otimes \mathcal{H}_E)$. Then, for any purification $\rho_{ABE} \in \mathcal{S}(\mathcal{H}_A \otimes \mathcal{H}_B \otimes \mathcal{H}_E)$ of ρ_{AE}, any Hilbert spaces $\mathcal{H}_{A'}$, $\mathcal{H}_{A''}$ and $\mathcal{H}_{E''}$ of dimension $d_{A'} = d_A$, $d_{A''} = d_A d_E$ and $d_{E''} = d_A^2$, respectively, any maximally entangled state $\Phi_{AA'} \in \Gamma_{AA'}$ and any pure state $|0\rangle\langle 0|_{E''} \in \mathcal{S}(\mathcal{H}_{E''})$, it holds that*

$$H_{\min}(A|E)_\rho = -\log d_A \max_{U_{E_{\text{tot}}}} \max_{\sigma_{BA''}} F^2(\Phi_{AA'} \otimes \sigma_{BA''}, (\mathbb{1}_{AB} \otimes U_{E_{\text{tot}}})\rho_{ABE_{\text{tot}}}(\mathbb{1}_{AB} \otimes U^\dagger_{E_{\text{tot}}})) \,, \tag{92}$$

where $\rho_{ABE_{\text{tot}}} = \rho_{ABE} \otimes |0\rangle\langle 0|_{E''}$ and where the first maximization ranges over unitaries

$$U_{E_{\text{tot}}} : \mathcal{H}_E \otimes \mathcal{H}_{E''} \to \mathcal{H}_{A'} \otimes \mathcal{H}_{A''} \,, \quad \text{where } \mathcal{H}_{A'} \otimes \mathcal{H}_{A''} \simeq \mathcal{H}_E \otimes \mathcal{H}_{E''} =: \mathcal{H}_{E_{\text{tot}}} \tag{93}$$

and the second maximization ranges over pure states $\sigma_{BA''} \in \mathcal{S}(\mathcal{H}_B \otimes \mathcal{H}_{A''})$.

Now we translate expression (92) into our generalized framework. We interpret the system E_{tot} as the system controlled by Eve, and therefore rename $E_{\text{tot}} \to E$.

- Since we want to arrive at an expression that does not make unnecessary assumptions about the mathematical description of the physical situation, we avoid the factor d_A present in (92). We look for a GPT analogue of $\max_{U_{E_{\text{tot}}}} \max_{\sigma_{BA''}} F^2(\Phi_{AA'} \otimes \sigma_{BA''}, (\mathbb{1}_{AB} \otimes U_{E_{\text{tot}}})\rho_{ABE_{\text{tot}}}(\mathbb{1}_{AB} \otimes U^\dagger_{E_{\text{tot}}}))$, omitting $-\log d_A$. As a consequence, we will have $H_{\min}(A|E)_\rho = -\log d_A \mathrm{Dec}(A|E)_\rho$ in quantum theory (see Example 25).
- We replace the maximization over all unitaries $U_{E_{\text{tot}}}$ acting on system E_{tot} by a supremum[7] over all local transformations $T_E \in \mathcal{T}_E$.[8]
- We generalize the quantum fidelity to the fidelity in abstract state spaces as defined in Definition 15.
- We replace the state $\rho_{ABE_{\text{tot}}} = \rho_{ABE} \otimes |0\rangle\langle 0|_{E''}$ by a state $\omega \in \Omega$.
- If we look at the state $\Phi_{AA'} \otimes \sigma_{BA''}$, we see that it is a state of maximal entanglement between Alice (A) and Eve ($A'A''$) in the sense that by performing measurements with elements of the form $P_A \otimes P_{A'} \otimes \mathbb{1}_B \otimes \mathbb{1}_{A''}$, they can get any statistics that two parties A and A' would be able to get by performing local measurements on the maximally entangled state $\Phi_{AA'}$. We translate this into our framework by assuming that there is a set Ψ_{AE} of "states with maximal correlation between Alice and Eve". Instead of minimizing over states $\Phi_{AA'} \otimes \sigma_{BA''}$, we then minimize over the set Ψ_{AE}.

We postpone the discussion of how such a set Ψ_{AE} looks like. We will give a definition of such a set further below. For now, we write down an expression for our decoherence quantity $\mathrm{Dec}(A|E)_\omega$ that depends on the choice of such a set $\Psi_{AE} \subseteq \Omega$. According to what we have just discussed, the expression is

$$\sup_{T_E \in \mathcal{T}_E} \sup_{\psi \in \Psi_{AE}} F^2(\psi, T_E(\omega)) \,. \tag{94}$$

We interpret the decoherence to be high when this quantity is high and vice versa, which is the opposite of $H_{\min}(A|E)_\rho$ (see the end of Supplementary Note 1). Before we can define $\mathrm{Dec}(A|E)_\omega$, however, we need to specify what a maximally entangled state in a GPT is.

---

[7] We do not assume enough about $\mathcal{T}_E$ to guarantee that the maximum is achieved, so we replace it by a supremum.

[8] One might raise the objection that in the quantum case, Example 18, the unitaries only correspond to those elements of $\mathcal{T}_E$ which bijectively map the space of density operators onto itself. It would be possible to include this restriction, but we decide not to do so, for two reason: We want to keep things simple, and we want to avoid the assumption that actions that the third party can perform can be purified as in the quantum case.

Definition of maximal correlation in GPTs

The expression (94) for our decoherence quantity $\mathrm{Dec}(A|E)_\omega$ contains a maximization over a set $\Psi_{AE} \subseteq \Omega$ which we interpret to be the set of states with maximal correlation between Alice and Eve. We now define this set.

Definition 23: *For a tripartite scenario S_{ABE}, we define the set Ψ_{AE} of* ***states with maximal correlation*** *between Alice and Eve by*

$$\Psi_{AE} := \left\{ \psi \in \Omega \;\middle|\; \begin{array}{l} \text{For every binary local instrument } I_A = \{T_A^0, T_A^1\} \in \mathcal{I}_A, \text{ there is a binary local} \\ \text{instrument } I_E = \{T_E^0, T_E^1\} \in \mathcal{I}_E \text{ such that } (u \circ T_A^0 \circ T_E^0)(\psi) + (u \circ T_A^1 \circ T_E^1)(\psi) = 1. \end{array} \right\}. \tag{95}$$

Definition 23 can be read as follows. The superscripts 0 and 1 of the elements of the instruments I_A and I_E stand for measurement outcomes, so $(u \circ T_A^0 \circ T_E^0)(\psi)$ or $(u \circ T_A^1 \circ T_E^1)(\psi)$ is the probability that Alice and Eve both get outcome 0 or both get outcome 1, respectively, when they measure with respect to I_A, I_E, respectively. Thus, the sum of these probabilities is the probability that Alice's and Eve's measurement outcomes are perfectly correlated. This means that for a state $\psi \in \Psi_{AE}$, it holds that for every binary measurement of Alice, there is a binary measurement for Eve such that their measurement outcomes are perfectly correlated.

A closer look at some subtleties is advisable here, both to avoid confusion and to see the advantages of the weak assumptions that define our framework. With reference to Example 22, one may point out that that the set

$$\left\{ \sigma \in \mathcal{S}(\mathcal{H}_A \otimes \mathcal{H}_B \otimes \mathcal{H}_E) \;\middle|\; \begin{array}{l} \text{For every binary POVM } \{P_A^0, P_A^1\} \text{ on } \mathcal{H}_A, \text{ there is a binary POVM} \\ \{P_E^0, P_E^1\} \text{ on } \mathcal{H}_E \text{ such that} \\ \qquad \mathsf{Tr}((P_A^0 \otimes \mathbb{1}_B \otimes P_E^0)\sigma) + \mathsf{Tr}((P_A^1 \otimes \mathbb{1}_B \otimes P_E^1)\sigma) = 1\,. \end{array} \right\} \tag{96}$$

is empty. This may seem to make our definition of Ψ_{AE} incompatible with quantum theory. Note, however, that the set

$$\left\{ \sigma \in \mathcal{S}(\mathcal{H}_A \otimes \mathcal{H}_B \otimes \mathcal{H}_E) \;\middle|\; \begin{array}{l} \text{For every binary projective measurement } \{P_A^0, P_A^1\} \text{ on } \mathcal{H}_A, \text{ there is a} \\ \text{binary projective measurement } \{P_E^0, P_E^1\} \text{ on } \mathcal{H}_E \text{ such that} \\ \qquad \mathsf{Tr}((P_A^0 \otimes \mathbb{1}_B \otimes P_E^0)\sigma) + \mathsf{Tr}((P_A^1 \otimes \mathbb{1}_B \otimes P_E^1)\sigma) = 1\,. \end{array} \right\} \tag{97}$$

is not empty as long as $\dim \mathcal{H}_E \geqslant \dim \mathcal{H}_A$. If $\mathcal{H}_E = \mathcal{H}_{A'} \otimes \mathcal{H}_{A''}$ with $\mathcal{H}_{A'} \simeq \mathcal{H}_A$, then this set contains all the states of the form $\Phi_{AA'} \otimes \sigma_{BA''}$ with $\Phi_{AA'} \in \Gamma_{AA'}$ as in (92). The advantage of our weak definition of the local transformations is that it does not force to see $\mathcal{T}_A$ as the analogue of the set of *all* CPMs of the form $\mathcal{R}_A \otimes \mathbb{1}_B \otimes \mathbb{1}_E$, but that it can be considered to be the analogue of all such CPMs which induce a functional of the form $\sigma \mapsto \mathsf{Tr}(P\sigma)$, where P is a projector. Example 22 can be modified accordingly (see Example 25 below). This makes our definition of Ψ_{AE} compatible with quantum theory.

With Definition 23 at hand, we are finally ready to define the decoherence quantity.

Definition 24: *Let S_{ABE} be a tripartite scenario, let $\omega \in \Omega$. We define the* ***decoherence quantity*** *of ω by*

$$\mathrm{Dec}(A|E)_\omega := \sup_{T_E \in \mathcal{T}_E} \sup_{\psi \in \Psi_{AE}} F^2(\psi, T_E(\omega)) \tag{98}$$

Example 25: *We consider a special case of a tripartite scenario in quantum theory. Consider*

$$((\mathrm{Herm}(\mathcal{H}), \mathrm{Pos}(\mathcal{H}), \mathsf{Tr}), \mathcal{T}_A, \mathcal{T}_B, \mathcal{T}_E)\,, \quad \textit{where} \tag{99}$$

$$\mathcal{H} = \mathcal{H}_A \otimes \mathcal{H}_B \otimes \mathcal{H}_E \tag{100}$$

$$\mathcal{T}_A = \left\{ \mathcal{R}_A \otimes \mathbb{1}_B \otimes \mathbb{1}_E \;\middle|\; \begin{array}{l} \mathcal{R}_A \textit{ is a trace non-increasing CPM on } \mathrm{Herm}(\mathcal{H}_A) \textit{ such that there is a} \\ \textit{projector } P_A \textit{ on } \mathcal{H}_A \textit{ with } \mathsf{Tr}(P_A\rho_A) = \mathsf{Tr}(\mathcal{R}_A(\rho_A)) \textit{ for all } \rho_A \in \mathcal{S}(\mathcal{H}_A) \end{array} \right\}, \tag{101}$$

and analogously for $\mathcal{T}_B$ and $\mathcal{T}_E$. In addition, we assume for simplicity that $\mathcal{H}_A \simeq \mathcal{H}_E$. In this case,

$$\Psi_{AE} = \left\{ \sigma \in \mathcal{S}(\mathcal{H}_A \otimes \mathcal{H}_B \otimes \mathcal{H}_E) \;\middle|\; \begin{array}{l} \text{For every binary projective measurement } \{P_A^0, P_A^1\} \text{ on } \mathcal{H}_A, \text{ there} \\ \text{is a binary projective measurement } \{P_E^0, P_E^1\} \text{ on } \mathcal{H}_E \text{ such that} \\ \qquad \mathsf{Tr}((P_A^0 \otimes \mathbb{1}_B \otimes P_E^0)\sigma) + \mathsf{Tr}((P_A^1 \otimes \mathbb{1}_B \otimes P_E^1)\sigma) = 1\,. \end{array} \right\} \tag{102}$$

$$= \{\Phi_{AE} \otimes \sigma_B \mid \Phi_{AE} \in \Gamma_{AE}\,,\ \sigma_B \in \mathcal{S}(\mathcal{H}_B)\}\,, \tag{103}$$

where Γ_{AE} is the set of maximally entangled states on $\mathcal{S}(\mathcal{H}_A \otimes \mathcal{H}_E)$ analogous to (15). For a pure state $\rho_{ABE} \in \mathcal{S}(\mathcal{H}_A \otimes \mathcal{H}_B \otimes \mathcal{H}_E)$, this gives us

$$\mathrm{Dec}(A|E)_\rho = \max_{\mathcal{R}_E} \max_{\Phi_{AE}} \max_{\sigma_B} F^2(\Phi_{AE} \otimes \rho_B, \mathbb{1}_A \otimes \mathbb{1}_B \otimes \mathcal{R}_E(\rho_{ABE})) \tag{104}$$

$$= \max_{\mathcal{R}_E} F^2(\Phi_{AE}, \mathbb{1}_A \otimes \mathcal{R}_E(\rho_{AE})) \tag{105}$$

$$= \frac{1}{d_A} 2^{-H_{\min}(A|E)_\rho} . \tag{106}$$

Hence, $H_{\min}(A|E)_\rho = -\log d_A \mathrm{Dec}(A|E)_\rho$. ■

Bounds on the decoherence quantity for GPTs

The goal of this subsection is to derive an upper bound on $\mathrm{Dec}(A|E)_\omega$ in terms of the CHSH winning probability of Alice and Bob. This is a practically relevant bound: On the premise that the channel behaves identically in multiple uses and does not build up correlations between different uses (such a channel is said to be *iid*, for *independent and identically distributed*), this winning probability can be estimated through repeated measurements on Alice's and Bob's side. What we show is that this estimate in turn gives a bound on $\mathrm{Dec}(A|E)_\omega$. In this section, we formulate this bound as a minimization problem which we solve and interpret in the last part of this Supplementary Note.

In the following, we derive a lower bound on $-\log \mathrm{Dec}(A|E)_\omega$. We make the convention that $-\log 0 = \infty$, where ∞ is a symbol for which we accept the inequality $\infty \geqslant r$ for every real number r. This lower bound on $-\log \mathrm{Dec}(A|E)_\omega$ then gives us an upper bound on $\mathrm{Dec}(A|E)_\omega$. In a first step, we bound the fidelity-based quantity $-\log \mathrm{Dec}(A|E)_\omega$ by a trace distance-based quantity. This has the advantage that the resulting optimization problems which give us the bounds can be solved using linear programming.

Proposition 26: *Let S_{ABE} be a tripartite scenario, let $\omega \in \Omega$. Then*

$$-\log \mathrm{Dec}(A|E)_\omega \geqslant \inf_{T_E \in \mathcal{T}_E} \inf_{\psi \in \Psi_{AE}} D^2(\psi, T_E(\omega)) . \tag{107}$$

The following lemma is useful for the proof of Proposition 26 below.

Lemma 27: *For all $x \in (0,1]$, it holds that $-\log(x^2) \geqslant 2(1-x)$.*

Proof. We have that $-\log(x^2) = -2\log(x)$, so the claim is equivalent to

$$(x-1) - \log(x) \geqslant 0 \quad \text{for all } x \in (0,1] . \tag{108}$$

The functions $F(x) = \log(x)$ and $G(x) = x - 1$ are differentiable on $\mathbb{R}_{>0}$. Thus, by the fundamental theorem of calculus, it holds that for all $x \in \mathbb{R}_{>0}$,

$$F(x) = F(1) + \int_1^x f(y)dy , \quad G(x) = G(1) + \int_1^x g(y)dy \quad \text{where} \quad f(y) = \frac{d}{dy}F(y) , \quad g(y) = \frac{d}{dy}G(y) , \tag{109}$$

so for all $x \in (0,1]$, we have that

$$(x-1) - \log(x) = G(x) - F(x) = \int_1^x g(y) - f(y)dy = -\int_x^1 \underbrace{1 - \frac{1}{\ln(2)y}}_{<0 \text{ for all } y \in (0,1]} dy \geqslant 0 . \tag{110}$$

This proves the claim. □

Proof of Proposition 26. Since the right hand side of (107) is a finite real number, the inequality trivially holds if $\mathrm{Dec}(A|E)_\omega = 0$ by the above convention. Thus, we assume in the following that $\mathrm{Dec}(A|E)_\omega > 0$. We have that

$$-\log \mathrm{Dec}(A|E)_\omega = -\log \sup_{T_E \in \mathcal{T}_E} \sup_{\psi \in \Psi_{AE}} F^2(\psi, T_E(\omega)) \tag{111}$$

$$= -\log \left(\sup_{T_E \in \mathcal{T}_E} \sup_{\psi \in \Psi_{AE}} F(\psi, T_E(\omega)) \right)^2 \tag{112}$$

For $x \in (0,1]$, it holds that $-\log x^2 \geqslant 2(1-x)$ (see Lemma 27). Thus, since

$$\sup_{T_E \in \mathcal{T}_E} \sup_{\psi \in \Psi_{AE}} F(\psi, T_E(\omega)) \in (0,1]\,, \tag{113}$$

we get that

$$\begin{aligned} -\log \mathrm{Dec}(A|E)_\omega &\geqslant 2\left(1 - \sup_{T_E \in \mathcal{T}_E} \sup_{\psi \in \Psi_{AE}} F(\psi, T_E(\omega))\right) & (114)\\ &= \inf_{T_E \in \mathcal{T}_E} \inf_{\psi \in \Psi_{AE}} 2(1 - F(\psi, T_E(\omega))) & (115)\\ &= \inf_{T_E \in \mathcal{T}_E} \inf_{\psi \in \Psi_{AE}} \sup_{M \in \mathcal{M}} 2(1 - b(\psi, T_E(\omega)|M))\,. & (116)\end{aligned}$$

For the Bhattacharyya coefficient b and the total variation distance d, it has been shown [41] that for any two probability distributions distributions, it holds that $2(1-b) \geqslant d^2$. Since this is true in particular for the two probability distributions that the measurement M induces on the states ψ and $T_E(\omega)$, we get that

$$\begin{aligned} -\log \mathrm{Dec}(A|E)_\omega &\geqslant \inf_{T_E \in \mathcal{T}_E} \inf_{\psi \in \Psi_{AE}} \sup_{M \in \mathcal{M}} d^2(\psi, T_E(\omega)|M) & (117)\\ &= \inf_{T_E \in \mathcal{T}_E} \inf_{\psi \in \Psi_{AE}} D^2(\psi, T_E(\omega))\,, & (118)\end{aligned}$$

as claimed. □

The idea that the fidelity and the trace distance are related is not new. In quantum theory, the *Fuchs-van de Graaf inequalities* (FvdG) relate the two quantities [42]. Inequality (107) is not completely analogous to the FvdG inequalities: It makes use of the logarithm in (107), which allows to apply classical relations that lead to a stronger bound than with the application of the FvdG inequalities.

For the bounds that we are going to derive, the notion of a *non-signalling distribution* is central. Our bounds are essentially minimizations of functions over sets of non-signalling distributions $\Pr[a,b,c|x,y,z]_\omega$ and $\Pr[a,c|x,z]_\psi$ with certain additional properties.

Definition 28: *A set of numbers* $\Pr[a,b,c|x,y,z]_\omega \in [0,1]$*, indexed by numbers* $a,b,c \in \{0,1\}$ *which we call* ***outcomes****, and numbers* $x,y,z \in \{0,1\}$ *which we call* ***settings****, is a* ***non-signalling distribution*** *if*

normalization:

$$\sum_{a,b,c} \Pr[a,b,c|x,y,z]_\omega = 1 \quad \textit{for all } x,y,z \in \{0,1\}\,, \tag{119}$$

no-signalling:

$$\begin{aligned} \sum_a \Pr[a,b,c|0,y,z]_\omega &= \sum_a \Pr[a,b,c|1,y,z]_\omega \quad \textit{for all } b,c,y,z \in \{0,1\}\,, & (120)\\ \sum_b \Pr[a,b,c|x,0,z]_\omega &= \sum_b \Pr[a,b,c|x,1,z]_\omega \quad \textit{for all } a,b,x,y \in \{0,1\}\,, & (121)\\ \sum_b \Pr[a,b,c|x,y,0]_\omega &= \sum_b \Pr[a,b,c|x,y,1]_\omega \quad \textit{for all } a,b,x,y \in \{0,1\}\,. & (122)\end{aligned}$$

Similarly, a set of numbers $\Pr[a,c|x,z]_\psi \in [0,1]$*, indexed by outcomes* $a,c \in \{0,1\}$ *and settings* $x,z \in \{0,1\}$ *is a non-signalling distribution if*

normalization:

$$\sum_{a,c} \Pr[a,c|x,z]_\psi = 1 \quad \textit{for all } x,z \in \{0,1\}\,, \tag{123}$$

no-signalling:

$$\begin{aligned} \sum_a \Pr[a,c|0,z]_\psi &= \sum_a \Pr[a,c|1,z]_\psi \quad \textit{for all } c,z \in \{0,1\}\,, & (124)\\ \sum_c \Pr[a,c|x,0]_\psi &= \sum_c \Pr[a,c|x,1]_\psi \quad \textit{for all } a,x \in \{0,1\}\,. & (125)\end{aligned}$$

The interpretation of equations (120) to (122) is that it is impossible for each of the three parties to *signal* to the other two parties by influencing their measurement statistics with the choice of the measurement setting. These one-party no-signalling constraints imply all the multi-party no-signalling constraints, saying that no collection of parties can signal to the remaining parties [43], so we do not need to require these constraints separately.

Now we are going to formulate the bound on $-\log \mathrm{Dec}(A|E)_\omega$ in terms of the CHSH winning probability of Alice and Bob. Assume that Alice, Bob and Eve are in a situation described by a tripartite scenario S_{ABE}. Suppose that Alice and Bob have estimated that for the state $\omega \in \Omega$ that they are analyzing, their CHSH winning probability is at least λ for some $\lambda \in [0,1]$. Formulated in our tripartite scenario language, this means that they have found out that for local instruments

$$I_A^0 = \{T_A^{0|0}, T_A^{1|0}\} \in \mathcal{I}_A, \qquad I_B^0 = \{T_B^{0|0}, T_B^{1|0}\} \in \mathcal{I}_B, \tag{126}$$

$$I_A^1 = \{T_A^{0|1}, T_A^{1|1}\} \in \mathcal{I}_A, \qquad I_B^1 = \{T_B^{0|1}, T_B^{1|1}\} \in \mathcal{I}_B\,, \tag{127}$$

it holds that

$$\frac{1}{4}\sum_{x,y}\sum_{\substack{a,b\\ a\oplus b=xy}} \left(u\circ T_A^{a|x}\circ T_B^{b|y}\right)(\omega) \geqslant \lambda\,. \tag{128}$$

In that case, what can Alice and Bob infer about $-\log \mathrm{Dec}(A|E)_\omega$? We have seen in Proposition 26 that this quantity is lower bounded by $\inf_{T_E\in\mathcal{T}_E}\inf_{\psi\in\Psi_{AE}} D^2(\psi, T_E(\omega))$. Alice's and Bob's estimate on their CHSH winning probability can be translated into a bound on this quantity. This is shown by the following proposition.

Proposition 29: *Let S_{ABE} be a tripartite scenario, let $\omega\in\Omega$ be a state. If the CHSH winning probability of Alice and Bob is at least λ, i.e. if there are local instruments I_A^0, I_A^1, I_B^0 and I_B^1 as in (126) and (127) and a $\lambda\in[0,1]$ such that (128) is satisfied, then*

$$\inf_{T_E\in\mathcal{T}_E}\inf_{\psi\in\Psi_{AE}} D(\psi, T_E(\omega)) \geqslant \min_{\substack{x,z\in\{0,1\}\\ \Pr[a,b,c|x,y,z]_\omega\in\mathcal{D}_\omega(\lambda)\\ \Pr[a,c|x,z]_\psi\in\mathcal{D}_\psi}} \frac{1}{2}\sum_{a,c}\left|\Pr[a,c|x,z]_\psi - \sum_b \Pr[a,b,c|x,y,z]_\omega\right|\,, \tag{129}$$

where $\mathcal{D}_\omega(\lambda)$ is the set of non-signalling distributions for Alice, Bob and Eve such that Alice and Bob have a CHSH winning probability of at least λ, i.e.

$$\mathcal{D}_\omega(\lambda) = \left\{ \Pr[a,b,c|x,y,z]_\omega \;\middle|\; \begin{array}{c} \Pr[a,b,c|x,y,z]_\omega \text{ is a non-signalling distribution such that} \\ \frac{1}{4}\sum_{x,y}\sum_{\substack{a,b\\ a\oplus b=xy}}\sum_c \Pr[a,b,c|x,y,z]_\omega \geqslant \lambda\,, \end{array} \right\}. \tag{130}$$

and where $\mathcal{D}_\psi$ is the set of non-signalling distributions for Alice and Eve such that their measurement outcomes are perfectly correlated when they choose the same measurement setting, i.e.

$$\mathcal{D}_\psi = \left\{ \Pr[a,c|x,z]_\psi \;\middle|\; \begin{array}{l} \Pr[a,c|x,z]_\psi \text{ is a non-signalling distribution} \\ \text{such that } \Pr[a=c|x=z]_\psi = 1. \end{array} \right\}. \tag{131}$$

Proposition 29 reduces our problem of lower bounding the decoherence quantity for GPTs to an optimization over non-signalling distributions. This allows us to use linear programming techniques, which in similar ways have been used in [44] to answer questions about non-signalling distributions.

We need the following lemma for the proof of Proposition 29 below.

Lemma 30: *Let S_{ABE} be a tripartite scenario, let $\omega,\psi\in\Omega$. Then, for all local instruments $(I_A, I_B, I_E)\in\mathcal{I}_A\times\mathcal{I}_B\times\mathcal{I}_E$, it holds that*

$$\inf_{T_E\in\mathcal{T}_E} D(\psi, T_E(\omega)) \geqslant \frac{1}{2}\inf_{T_E\in\mathcal{T}_E}\sum_{\substack{T_A\in I_A\\ U_E\in I_E}}\left|(u\circ T_A\circ U_E)(\psi) - \sum_{T_B\in I_B}(u\circ T_A\circ T_B\circ U_E\circ T_E)(\omega)\right|\,. \tag{132}$$

Proof. It is sufficient to show that for all $\omega,\psi\in\Omega$, for all $T_E\in\mathcal{T}_E$ and for all $(I_A,I_B,I_E)\in\mathcal{I}_A\times\mathcal{I}_B\times\mathcal{I}_E$,

$$D(\psi, T_E(\omega)) \geqslant \frac{1}{2}\sum_{\substack{T_A\in I_A\\ U_E\in I_E}}\left|(u\circ T_A\circ U_E)(\psi) - \sum_{T_B\in I_B}(u\circ T_A\circ T_B\circ U_E\circ T_E)(\omega)\right|\,. \tag{133}$$

This is what we are going to show now. Let $\omega, \psi \in \Omega$, let $T_E \in \mathcal{T}_E$, let $(I_A, I_B, I_E) \in \mathcal{I}_A \times \mathcal{I}_B \times \mathcal{I}_E$. Then

$$D(\psi, T_E(\omega)) = \sup_{M \in \mathcal{M}} d(\psi, T_E(\omega)|M) = \frac{1}{2} \sup_{M \in \mathcal{M}} \sum_{e \in M} |e(\psi) - e(T_E(\omega))| \, . \tag{134}$$

If instead of taking the supremum over $\mathcal{M}$, we only evaluate the expression for a particular element of $\mathcal{M}$, we get a lower bound on (134). We choose the element (c.f. Remark 21)

$$\{u \circ T_A \circ U_E \mid T_A \in \mathcal{I}_A, U_E \in \mathcal{I}_E\} \in \mathcal{M} \, . \tag{135}$$

Hence,

$$D(\psi, T_E(\omega)) \geqslant \frac{1}{2} \sum_{\substack{T_A \in \mathcal{I}_A \\ U_E \in \mathcal{I}_E}} |(u \circ T_A \circ U_E)(\psi) - (u \circ T_A \circ U_E \circ T_E)(\omega)| \, . \tag{136}$$

By the definition of a local instrument, $u = \sum_{T_B \in I_B} u \circ T_B$. Thus,

$$D(\psi, T_E(\omega)) \geqslant \frac{1}{2} \sum_{\substack{T_A \in \mathcal{I}_A \\ U_E \in \mathcal{I}_E}} \left| (u \circ T_A \circ U_E)(\psi) - \sum_{T_B \in I_B} (u \circ T_B \circ T_A \circ U_E \circ T_E)(\omega) \right| \tag{137}$$

$$= \frac{1}{2} \sum_{\substack{T_A \in \mathcal{I}_A \\ U_E \in \mathcal{I}_E}} \left| (u \circ T_A \circ U_E)(\psi) - \sum_{T_B \in I_B} (u \circ T_A \circ T_B \circ U_E \circ T_E)(\omega) \right| \, , \tag{138}$$

where in the last equality, we made use of the fact that transformations of different parties commute. □

Proof of Proposition 29. It is sufficient to show that for every $\psi \in \Psi_{AE}$, the claimed inequality holds without the minimization over Ψ_{AE}, i.e.

$$\inf_{T_E \in \mathcal{T}_E} D(\psi, T_E(\omega)) \geqslant \min_{\substack{x,z \in \{0,1\} \\ \Pr[a,b,c|x,y,z]_\omega \in \mathcal{D}_\omega(\lambda) \\ \Pr[a,c|x,z]_\psi \in \mathcal{D}_\psi(\Psi_{AE}, \lambda)}} \frac{1}{2} \sum_{a,c} \left| \Pr[a,c|x,z]_\psi - \sum_b \Pr[a,b,c|x,y,z]_\omega \right| \, . \tag{139}$$

By means of Lemma 30, we know that for all $x, y \in \{0,1\}$ and every $I_E \in \mathcal{I}_E$,

$$\inf_{T_E \in \mathcal{T}_E} D(\psi, T_E(\omega)) \geqslant \frac{1}{2} \inf_{T_E \in \mathcal{T}_E} \sum_{\substack{a \in \{0,1\} \\ U_E \in I_E}} \left| (u \circ T_A^{a|x} \circ U_E)(\psi) - \sum_{b \in \{0,1\}} (u \circ T_A^{a|x} \circ T_B^{b|y} \circ U_E \circ T_E)(\omega) \right| \, . \tag{140}$$

Let $\psi \in \Psi_{AE}$, let $I_E^0 = \{U_E^{0|0}, U_E^{1|0}\}$, $I_E^1 = \{U_E^{0|1}, U_E^{1|1}\} \in \mathcal{I}_E$ be local instruments for Eve such that

$$(u \circ T_A^{0|0} \circ U_E^{0|0})(\psi) + (u \circ T_A^{1|0} \circ U_E^{1|0})(\psi) = 1 \, , \tag{141}$$

$$(u \circ T_A^{0|1} \circ U_E^{0|1})(\psi) + (u \circ T_A^{1|1} \circ U_E^{1|1})(\psi) = 1 \, , \tag{142}$$

which exist according to the definition of Ψ_{AE} (Definition 23). It holds that for every $x, y, z \in \{0,1\}$,

$$\inf_{T_E \in \mathcal{T}_E} D(\psi, T_E(\omega)) \geqslant \frac{1}{2} \inf_{T_E \in \mathcal{T}_E} \sum_{a,c \in \{0,1\}} \left| (u \circ T_A^{a|x} \circ U_E^{c|z})(\psi) - \sum_{b \in \{0,1\}} (u \circ T_A^{a|x} \circ T_B^{b|y} \circ U_E^{c|z} \circ T_E)(\omega) \right| \, . \tag{143}$$

$$= \frac{1}{2} \inf_{T_E \in \mathcal{T}_E} \sum_{a,c \in \{0,1\}} \left| \Pr[a,c|x,z]_\psi - \sum_{b \in \{0,1\}} \Pr[a,b,c|x,y,z]_\omega \right| \, , \tag{144}$$

where

$$\Pr[a,c|x,z]_\psi = (u \circ T_A^{a|x} \circ U_E^{c|z})(\psi) \, , \tag{145}$$

$$\Pr[a,b,c|x,y,z]_\omega = (u \circ T_A^{a|x} \circ T_B^{b|y} \circ U_E^{c|z} \circ T_E)(\omega) \, . \tag{146}$$

Hence,

$$\inf_{T_E\in\mathcal{T}_E} D(\psi,T_E(\omega)) \geqslant \frac{1}{2}\min_{x,y,z}\inf_{T_E\in\mathcal{T}_E}\sum_{a,c\in\{0,1\}}\left|\Pr[a,c|x,z]_\psi - \sum_{b\in\{0,1\}}\Pr[a,b,c|x,y,z]_\omega\right| . \tag{147}$$

$\Pr[a,c|x,z]_\psi$ forms a non-signalling distribution: For the normalization, note that for all $x,z\in\{0,1\}$, we have that

$$\sum_{a,c}\Pr[a,c|x,z]_\psi = \sum_{a,c}(u\circ T_A^{a|x}\circ U_E^{c|z})(\psi) = \underbrace{\left(\sum_a u\circ T_A^{a|x}\right)}_{u}\circ\left(\sum_c U_E^{c|z}\right)(\psi) \tag{148}$$

$$= \left(\sum_c u\circ U_E^{c|z}\right)(\psi) = u(\psi) = 1\,. \tag{149}$$

For the no-signalling condition, note that for all $c,z\in\{0,1\}$, it holds that

$$\sum_a \Pr[a,c|0,z]_\psi = \left(\left(\sum_a u\circ T_A^{a|0}\right)\circ U_E^{c|z}\right)(\psi) = (u\circ U_E^{c|z})(\psi) = \left(\left(\sum_a u\circ T_A^{a|1}\right)\circ U_E^{c|z}\right)(\psi) \tag{150}$$

$$= \sum_a \Pr[a,c|1,z]_\psi\,, \tag{151}$$

and that for all $a,x\in\{0,1\}$, it holds that

$$\sum_c \Pr[a,c|x,0]_\psi = \sum_c (u\circ T_A^{a|x}\circ U_E^{c|0})(\psi) = \sum_c (u\circ U_E^{c|0}\circ T_A^{a|x})(\psi) = \left(\left(\sum_c u\circ U_E^{c|0}\right)\circ T_A^{a|x}\right)(\psi) \tag{152}$$

$$= (u\circ T_A^{a|x})(\psi) = \left(\left(\sum_c u\circ U_E^{c|1}\right)\circ T_A^{a|x}\right)(\psi) = \sum_c \Pr[a,c|x,1]_\psi\,, \tag{153}$$

where in the second equality, we made use of the fact that local transformations of different parties commute. Analogously, for every $T_E\in\mathcal{T}_E$, $\Pr[a,b,c|x,y,z]_\omega$ is a non-signalling distribution. Moreover, $\Pr[a,b,c|x,y,z]_\omega$ satisfies

$$\frac{1}{4}\sum_{x,y}\sum_{\substack{a,b\\a\oplus b=xy}}\sum_c \Pr[a,b,c|x,y,z]_\omega = \frac{1}{4}\sum_{x,y}\sum_{\substack{a,b\\a\oplus b=xy}}\sum_c (u\circ T_A^{a|x}\circ T_B^{b|y}\circ U_E^{c|z}\circ T_E)(\omega) \tag{154}$$

$$= \frac{1}{4}\sum_{x,y}\sum_{\substack{a,b\\a\oplus b=xy}}\underbrace{\left(\sum_c (u\circ U_E^{c|z}\circ T_E\right)}_{u}\circ T_A^{a|x}\circ T_B^{b|y})(\omega) \tag{155}$$

$$\geqslant \lambda\,, \tag{156}$$

where the inequality is one of the assumptions of the proposition. Furthermore, $\Pr[a,c|x,1]_\psi$ satisfies

$$\Pr[0,0|0,0]_\psi + \Pr[1,1|0,0]_\psi = (u\circ T_A^{0|0}\circ U_E^{0|0})(\psi) + (u\circ T_A^{1|0}\circ U_E^{1|0})(\psi) = 1\,, \tag{157}$$

$$\Pr[0,0|1,1]_\psi + \Pr[1,1|1,1]_\psi = (u\circ T_A^{0|1}\circ T^{0|1})(\psi) + (u\circ T_A^{1|1}\circ U_E^{1|1})(\psi) = 1 \tag{158}$$

(where we made use of (141) and (142)), which we may abbreviate as $\Pr[a=c|x=z]_\psi = 1$. Thus, for every $T_E\in\mathcal{T}_E$, we have that $\Pr[a,b,c|x,y,z]_\omega\in\mathcal{D}_\omega(\lambda)$ and $\Pr[a,c|x,z]_\psi\in\mathcal{D}_\psi$. Thus,

$$\inf_{T_E\in\mathcal{T}_E} D(\psi,T_E(\omega)) \geqslant \frac{1}{2}\min_{x,y,z}\inf_{\substack{\Pr[a,b,c|x,y,z]_\omega\in\mathcal{D}_\omega(\lambda)\\ \Pr[a,c|x,z]_\psi\in\mathcal{D}_\psi(\Psi_{AE},\lambda)}}\sum_{a,c\in\{0,1\}}\left|\Pr[a,c|x,z]_\psi - \sum_{b\in\{0,1\}}\Pr[a,b,c|x,y,z]_\omega\right| . \tag{159}$$

Since $\Pr[a,b,c|x,y,z]_\omega$ satisfies the no-signalling property, the right hand side of (159) is independent of y, so the minimization only needs to be performed over x and z. Moreover, the infimum over the sets $\mathcal{D}_\omega(\lambda)$ and $\mathcal{D}_\psi$ is a minimum because it is the infimum of a continuous function over a convex polytope, which is always attained (see the last part of this Supplementary Note for more details). This completes the proof. □

Corollary 31 (The bound)**:** *Let S_{ABE} be a tripartite scenario, let $\omega \in \Omega$ be a state. If the CHSH winning probability of Alice and Bob is at least λ (in the above sense), then*

$$\mathrm{Dec}(A|E)_\omega \leqslant 2^{-\delta^2(\lambda)}\,, \tag{160}$$

where

$$\delta(\lambda) = \min_{\substack{x,z\in\{0,1\} \\ \Pr[a,b,c|x,y,z]_\omega\in\mathcal{D}_\omega(\lambda) \\ \Pr[a,c|x,z]_\psi\in\mathcal{D}_\psi}} \frac{1}{2}\sum_{a,c}\left|\Pr[a,c|x,z]_\psi - \sum_b \Pr[a,b,c|x,y,z]_\omega\right|\,, \tag{161}$$

Proof. This is a direct consequence of Propositions 26 and 29. □

Evaluation of the bound and results

Formulation of the bound as a linear program

In this subsection, we evaluate the bound (160). To this end, we rewrite (161) in terms of linear programs.

Linear Program: *The bound $\delta(\lambda)$, which is a function $\delta : [0,1] \to [0,1]$, is given as follows. For all $\lambda \in [0,1]$, the value $\delta(\lambda)$ is the solution of the linear program*

$$\begin{array}{ll} \textit{minimize} & \delta(\lambda) \\ \textit{subject to} & \Pr[a,b,c|x,y,z]_\omega \in \mathcal{D}_\omega(\lambda) \\ & \Pr[a,c|x,z]_\psi \in \mathcal{D}_\psi \\ & \delta(\lambda) \geqslant \sum_{a,c}\delta^{xz}_{ac}\ \forall x,z\in\{0,1\} \\ & \delta^{xz}_{ac} \geqslant \frac{1}{2}\left(\Pr[a,c|x,z]_\psi - \sum_b \Pr[a,b,c|x,0,z]_\omega\right) \geqslant -\delta^{xz}_{ac}\ \forall a,c,x,z\in\{0,1\} \end{array} \tag{162}$$

This is a linear program in 97 variables:

$$\begin{array}{lr} \left\{\Pr[a,b,c|x,y,z]_\omega\right\}_{a,b,c,x,y,z\in\{0,1\}} & 64 \text{ variables} \\ \left\{\Pr[a,c|x,z]_\psi\right\}_{a,c,x,z\in\{0,1\}} & +\,16 \text{ variables} \\ \left\{\delta^{xz}_{ac}\right\}_{a,c,x,z\in\{0,1\}} & +\,16 \text{ variables} \\ \delta(\lambda) & +\,1 \text{ variable} \\ & =97 \text{ variables} \end{array}$$

We have already written out the constraints for these 97 variables as (in)equalities. The third and fourth line are already written as such in the program description, and for the first two lines, we refer to the following:

constraint	(in)equalities
$\Pr[a,b,c|x,y,z]_\omega \in \mathcal{D}_\omega(\lambda)$	(119) to (122) and inequality in (130)
$\Pr[a,c|x,z]_\psi \in \mathcal{D}_\psi(\lambda)$	(123) to (125) and equation in (131)

The inequalities define a convex polytope over which the convex function $\delta(\lambda)$ is minimized, so the minimum is attained. It is straightforward to bring these inequalities into the standard form of linear programming. We solved the resulting linear program using standard linear programming routines in Mathematica and Octave.

Solution of the linear program and discussion of the results

We plot the result in Supplementary Fig. 8. The bound $2^{-\delta^2(\lambda)}$ is non-trivial for values $\lambda \in (3/4,1]$. This is a very satisfactory result as one cannot expect the bound to be non-trivial for $\lambda \in [0,3/4]$: A CHSH winning probability of at least $\lambda \in [0,3/4]$ for Alice and Bob is always compatible with $\mathrm{Dec}(A|E)_\omega = 1$. To see this, note that the requirement for a state to yield a CHSH winning probability for Alice and Bob of at least $\lambda \in [0,3/4]$ is trivial: Alice and Bob can choose trivial measurements that always yields 1 as an outcome, independently of the state. More precisely, in our tripartite scenario language, we can express this as follows. Certainly, there are tripartite scenarios in which the

identity map $\mathbb{1}_V$ and the zero map 0_V are in $\mathcal{T}_A$, $\mathcal{T}_B$ and $\mathcal{T}_E$.[9] For such tripartite scenarios, the condition (c.f. (126) to (128))

$$\frac{1}{4}\sum_{x,y}\sum_{\substack{a,b\\a\oplus b=xy}}\left(u\circ T_A^{a|x}\circ T_B^{b|y}\right)(\omega)\geqslant\lambda\in[0,3/4]\quad\text{for some}\quad\begin{matrix}\{T_A^{0|0},T_A^{1|0}\},\{T_A^{0|1},T_A^{1|1}\}\in\mathcal{I}_A,\\\{T_B^{0|0},T_B^{1|0}\},\{T_B^{0|1},T_B^{1|1}\}\in\mathcal{I}_B\end{matrix}\tag{163}$$

is always satisfied because for all $\omega\in\Omega$,

$$\frac{1}{4}\sum_{x,y}\sum_{\substack{a,b\\a\oplus b=xy}}\left(u\circ T_A^{a|x}\circ T_B^{b|y}\right)(\omega)=\frac{3}{4}\quad\text{for}\quad\begin{matrix}T_A^{0|0}=T_A^{0|1}=T_B^{0|0}=T_B^{0|1}=0_V\,,\\T_A^{1|0}=T_A^{1|1}=T_B^{1|0}=T_B^{1|1}=\mathbb{1}_V\,.\end{matrix}\tag{164}$$

This means that the requirement that the CHSH winning probability for Alice and Bob is at least $\lambda\in[0,3/4]$ does not exclude the case $\omega\in\Psi_{AE}$. In that case, $\mathrm{Dec}(A|E)_\omega=\sup_{T_E\in\mathcal{T}_E}\sup_{\psi\in\Psi_{AE}}F^2(\psi,T_E(\omega))=1$.

[9] Every tripartite scenario can be turned into such by adding $\mathbb{1}_V$ and 0_V to the sets of local transformations. In fact, it would be physical to assume that each set of local transformations contains $\mathbb{1}_V$ and 0_V.

Supplementary Note 4: General approach for the application of the decoherence formalism

In the previous part of this Supplementary Information, we developed our decoherence estimation formalism. In this part, we demonstrate how our formalism can be used to devise tests for given models of decoherence. We will calculate the decoherence quantity for some standard models of decoherence and for a model of gravitational decoherence in Supplementary Note 5, and we will derive a full test for a model of gravitational decoherence in Supplementary Note 6.

We emphasize that the material presented in Supplementary Notes 5 and 6 constitutes *example* applications of our formalism. We want to see our formalism in action for some examples of decoherence models, and how one can derive a test for a given model. However, our formalism is universal, and its application is not limited to the particular models considered in Supplementary Notes 5 and 6. Therefore, before diving into the applications, we want to give a general picture for how to derive a test from our formalism for a given model for (gravitational) decoherence.

In the literature, there are lots of different theories about how gravitational decoherence affects physical systems. Many of them eventually describe gravitational decoherence as an evolution of a quantum system that is coupled to another system (see the dark gray part in Fig. 1 of the main article). This is effectively describing gravitational decoherence as a quantum channel. In order to test such theories, one would perform measurements on the affected system to see whether the outcomes are compatible with the tested theory. Such a theory and its test rely on the assumption that quantum theory is a correct description for the affected system and the gravitational decoherence process that it undergoes.

Our decoherence estimation formalism allows to take such a theory and turn its test into a *universal* test which does not rely on the validity of quantum theory (see the light gray part in Fig. 1 of the main article). In this universal test, the system undergoing gravitational decoherence is part of a maximally correlated pair. Measurements on both parts of this maximally entangled pair allow to estimate the CHSH parameter β, which in turn yields a bound $h(\beta)$ on the decoherence that took place in the experiment. This bound $h(\beta)$ can be formulated in any theory in which there is a notion of a maximally correlated pair, rather than just quantum theory.

To reach at such a universal test, some work needs to be done, which is divided into two parts. Firstly, one needs to decide on the range of theories within which one wants to test. The range of theories is given by the constrants that one imposes on the theories. Different constraints lead to different functions h that relate the measured parameter β to the bound on the decoherence of the process. We have seen two extreme cases of such constraints: In Supplementary Note 2, we have derived h for the case where the constraints are given by quantum theory, in which case we get

$$h(\beta) = \frac{1}{d_A} 2^{-f(\beta)} \tag{165}$$

with f given in equation (19). The other extreme case that we look at is the case where the only constraint is given by the no-signalling property. This leads to a test within a much larger range of theories. As we have seen, the bound that we get in that case is given by Equation (160) and Supplementary Fig. 8. However, these are just two out of many possible ranges of theories within which one can test decoherence processes with our framework.

Secondly, one needs to determine how the existing theory affects one half of a *pair* of systems. Then, given an input state of the pair, one can calculate $\mathrm{Dec}(A|E)$ for this pair. This will give a value for the decoherence quantity that one can test in the experiment.

The first part (determining h) is what we demonstrated in Supplementary Notes 2 and 3. In Supplementary Notes 5 and 6, we will demonstrate how the second part is done. In Supplementary Note 5, we calculate $\mathrm{Dec}(A|E)$ for the case where the input state of the pair is the maximally entangled state

$$|\Phi^+\rangle_{AA'} = \frac{1}{\sqrt{2}} \left(|0\rangle_A \otimes |0\rangle_{A'} + |1\rangle_A \otimes |1\rangle_{A'} \right) , \tag{166}$$

which leads to the final state

$$\rho_{AB}\left(\Lambda\right) = (\mathbb{1}_A \otimes \Lambda_{A' \to B}) |\Phi^+\rangle\langle\Phi^+|_{AA'} , \tag{167}$$

The state $\rho_{AB}\left(\Lambda\right)$ as in (167) is known in the literature as the *Choi representation* of the channel Λ (c.f. *channel-state-duality*). Written out as a matrix in the product computational basis, the state $\rho_{AB}\left(\Lambda\right)$ becomes the *Choi matrix* of the channel. The decoherence quantity is then a function of its Choi representation, namely

$$\mathrm{Dec}(A|E)_{\rho(\Lambda)} = \max_{\mathcal{R}_{E \to A'}} F^2(\Phi_{AA'}, \mathbb{1}_A \otimes \mathcal{R}_{E \to A'}(\rho_{AB}\left(\Lambda\right))) , \quad \text{where} \quad \rho_{ABE}(\Lambda) \text{ purifies } \rho_{AB}\left(\Lambda\right) . \tag{168}$$

These are calculations within quantum theory, which are just here to show the relation between a decoherence model (which in this case is a quantum channel) and the decoherence quantity associated with it. We emphasize again

that this part can alternatively be done in any probabilistic theory. Some of our calculations also concern models for gravitational decoherence that have been considered in the literature.

In Supplementary Note 6, we derive a full test for a model for gravitational decoherence. We consider a model first proposed by Bouwmeester [45]. It is a model that describes a photon in an optomechanical cavity. We extend this model where two entangled photons are located in two different cavities, one of which is coupled to a movable mirror introducing gravitational decoherence. This way, we extend the single-photon experiment to a two-photon experiment that can be tested in our formalilsm. We calculate $\mathrm{Dec}(A|E)$ for this experiment. Then, we calculate the minimal β that needs to be measured in order to failsify this model, for the case where h is chosen to be the quantum bound. We emphasize, however, that this choice of the bound can be modified within our framework in order to arrive at tests for other ranges of theories. The parameter β measured in the experiment can be reconsidered in all possible ranges of theories.

Supplementary Note 5: The decoherence quantity for some models of decoherence

In this appendix, we calculate the decoherence quantity for some important standard classes of channels, for some of which we give closed-form expressions. As we will see, some models in the literature predict that gravitational decoherence acts like one of these standard channels. This allows us to derive closed-form expressions for the predicted values of the decoherence quantity for some models for gravitational decohrence.

More precisely, we calculate the decoherence quantity for the following channels:

- Pauli channels, including the special cases
  - depolarizing channel,
  - dephasing channel,
  - 2-Pauli channel,
- gravitational decoherence due to metric fluctuations in flat space time [46], which we will reduce to the decoherence of a Pauli channel,
- amplitude damping channel,
- erasure channel.

In each of these cases, we look at the state that results from the action of the channel on one half of a maximally entangled state, equation (167), and calculate its decoherence quantity, (168). This quantity can be calculated as

$$\mathrm{Dec}(A|E)_{\rho(\Lambda)} = \frac{1}{d_A} 2^{-H_{\min}(A|E)_\rho} \tag{169}$$

$$= \frac{1}{d_A} 2^{H_{\max}(A|B)_\rho} \tag{170}$$

where we got the first equality from equation (106) and the second equality from Lemma 6. Hence, calculating the decoherence quantity for a channel reduces to calculating the max-entropy of its Choi matrix. We will make use of this in all of the following examples.

The decoherence quantity for Pauli channels

Here we consider a class of qubit-to-qubit channels which is called the class of *Pauli channels*. Many decoherence models turn out to be Pauli channels or turn out to have the same strength of decoherence as the Pauli channels. We will see two examples for that: our calculations in the next subsection and in Supplementary Note 6 will reduce the gravitational decoherence of some models to the decoherence of Pauli channels.

The Pauli channels get their names from the fact that they correspond to the probabilistic application of the Pauli operators σ_x, σ_y and σ_z. They are defined as follows.

Definition 32: *For probabilities $p_x, p_y, p_z \in [0,1]$ with $p_x + p_y + p_y \leqslant 1$, the* **Pauli channel** $C^{\mathrm{Pauli}}_{\vec{p}}$ *with respect to $\vec{p} = (p_x, p_y, p_z)$ is defined as the channel*

$$\begin{aligned} C^{\mathrm{Pauli}}_{\vec{p}} : \ \mathcal{S}(\mathbb{C}^2) \ &\to \ \mathcal{S}(\mathbb{C}^2)\,, \\ \rho \ &\mapsto \ (1-p_x-p_y-p_z)\rho + p_x\sigma_x\rho\sigma_x + p_y\sigma_y\rho\sigma_y + p_z\sigma_z\rho\sigma_z\,. \end{aligned} \tag{171}$$

It is easy to calculate that in the computational basis (in which σ_z is diagonal), the Choi Matrix (c.f. Supplementary Note 4) of the Pauli channel $C_{\vec{p}}^{\text{Pauli}}$ is given by

$$\rho_{AB}(C_{\vec{p}}^{\text{Pauli}}) = (\mathbb{1} \otimes C_{\vec{p}}^{\text{Pauli}})|\Phi^+\rangle\langle\Phi^+| = \begin{pmatrix} 1-p_x-p_y & 0 & 0 & 1-p_x-p_y-2p_z \\ 0 & p_x+p_y & p_x-p_y & 0 \\ 0 & p_x-p_y & p_x+p_y & 0 \\ 1-p_x-p_y-2p_z & 0 & 0 & 1-p_x-p_y \end{pmatrix} . \tag{172}$$

It turns out that the state $\rho_{AB}(C_{\vec{p}}^{\text{Pauli}})$ is Bell-diagonal. Consider the Bell basis $(|\Psi^-\rangle, |\Psi^+\rangle, |\Phi^-\rangle, |\Phi^+\rangle)$, where

$$|\Psi^-\rangle = \frac{1}{\sqrt{2}}(|01\rangle - |10\rangle) , \qquad |\Phi^-\rangle = \frac{1}{\sqrt{2}}(|00\rangle - |11\rangle) , \tag{173}$$

$$|\Psi^+\rangle = \frac{1}{\sqrt{2}}(|01\rangle + |10\rangle) , \qquad |\Phi^+\rangle = \frac{1}{\sqrt{2}}(|00\rangle + |11\rangle) . \tag{174}$$

In this basis,

$$\rho_{AB}(C_{\vec{p}}^{\text{Pauli}})_{\text{Bell}} = \begin{pmatrix} p_y & 0 & 0 & 0 \\ 0 & p_x & 0 & 0 \\ 0 & 0 & p_z & 0 \\ 0 & 0 & 0 & 1-p_x-p_y-p_z \end{pmatrix} . \tag{175}$$

This allows us to apply Lemma 9 in order to get

$$\text{Dec}(A|E)_{\rho(C_{\vec{p}}^{\text{Pauli}})} = \frac{1}{d_A} 2^{H_{\max}(A|B)_{\rho(C_{\vec{p}}^{\text{Pauli}})}} , \tag{176}$$

$$= \frac{1}{2} 2^{-\log 2 + 2\log\left(\sqrt{p_x}+\sqrt{p_y}+\sqrt{p_z}+\sqrt{1-p_x-p_y-p_z}\right)} \tag{177}$$

$$= \frac{1}{4}\left(\sqrt{p_x}+\sqrt{p_y}+\sqrt{p_z}+\sqrt{1-p_x-p_y-p_z}\right)^2 . \tag{178}$$

Expression (178) allows us to calculate the decoherence quantity for various special cases of Pauli channels. We consider the following four special cases, each of which is a one-parameter subfamily of the Pauli channels.

- For $p \in [0,1]$, the *depolarizing channel* of strength p is given by

$$C^{\text{Pauli}}_{(p/4,p/4,p/4)}(\rho) = \left(1 - \frac{3p}{4}\right)\rho + \frac{p}{4}\sigma_x\rho\sigma_x + \frac{p}{4}\sigma_y\rho\sigma_y + \frac{p}{4}\sigma_z\rho\sigma_z \tag{179}$$

$$= (1-p)\rho + p\frac{\mathbb{1}}{2} , \tag{180}$$

  and its decoherence quantity is given by

$$\text{Dec}(A|E)_{\rho(C^{\text{Pauli}}_{(p/4,p/4,p/4)})} = \frac{1}{4}\left(\frac{3\sqrt{p}}{2} + \sqrt{1-\frac{3p}{4}}\right)^2 . \tag{181}$$

- For $p \in [0,1]$, the *dephasing channel* of strength p is given by[10]

$$C^{\text{Pauli}}_{(0,0,p/2)}(\rho) = \left(1 - \frac{p}{2}\right)\rho + \frac{p}{2}\sigma_z\rho\sigma_z , \tag{182}$$

  and its decoherence quantity is given by

$$\text{Dec}(A|E)_{\rho(C^{\text{Pauli}}_{(0,0,p/2)})} = \frac{1}{4}\left(\sqrt{\frac{p}{2}} + \sqrt{1-\frac{p}{2}}\right)^2 . \tag{183}$$

  The dephasing channel in Y, $C^{\text{Pauli}}_{(0,p/2,0)}$ and the dephasing channel in Z, $C^{\text{Pauli}}_{(0,0,p/2)}$, both have the same decoherence quantity as the dephasing channel, (183).

---

[10] We choose to parametrize the Pauli channels such that they have their strongest decoherence for $p = 1$, so that they are all comparable.

- For $p \in [0,1]$, the *2-Pauli channel in X and Y* of strength p is given by

$$C^{\text{Pauli}}_{(p/3,p/3,0)}(\rho) = \left(1 - \frac{2p}{3}\right)\rho + \frac{p}{3}\sigma_x\rho\sigma_x + \frac{p}{3}\sigma_y\rho\sigma_y\,, \tag{184}$$

and its decoherence quantity is given by

$$\text{Dec}(A|E)_{\rho(C^{\text{Pauli}}_{(p/3,p/3,0)})} = \frac{1}{4}\left(2\sqrt{\frac{p}{3}} + \sqrt{1 - \frac{2p}{3}}\right)^2 . \tag{185}$$

The other two 2-Pauli channels, $C^{\text{Pauli}}_{(p/3,0,p/3)}$ and $C^{\text{Pauli}}_{(0,p/3,p/3)}$, have the same decoherence quantity, (185).

- For $p \in [0,1]$, the *BB84 channel* of strength p is given by

$$C^{\text{Pauli}}_{((p/2)(1-p/2),(1-p/2)^2),(p/2)(1-p/2)}(\rho) = \left(\frac{p}{2}\right)^2\rho + \frac{p}{2}\left(1 - \frac{p}{2}\right)\sigma_x\rho\sigma_x + \left(1 - \frac{p}{2}\right)^2\sigma_y\rho\sigma_y\,, + \frac{p}{2}\left(1 - \frac{p}{2}\right)\sigma_z\rho\sigma_z\,, \tag{186}$$

and its decoherence quantity is given by

$$\text{Dec}(A|E)_{\rho(C^{\text{Pauli}}_{(p/3,p/3,0)})} = \frac{1}{4}\left(1 + 2\sqrt{\frac{p}{2}\left(1 - \frac{p}{2}\right)}\right)^2 . \tag{187}$$

A plot of $\text{Dec}(A|E)_{\rho(C^{\text{Pauli}}_{\vec{p}})}$ for these four special cases of Pauli channels is shown in Supplementary Fig. 9 as a function of the strength parameter p. We have chosen the parametrizations such that the channel is the identity channel for $p = 0$ and has its strongest decoherence for $p = 1$, so that the channels are comparable.

Example application of Pauli decoherence to a model for gravitational decoherence

In this subsection, we apply the knowledge about the decoherence of Pauli channels that we gained in the last subsection to derive a closed-form expression for the decoherence quantity for some gravitational decoherence channel that has been suggested in the literature. We emphasize that the calculations presented here are only one example application of our decoherence formalism. Many other models for gravitational decoherence or other decoherence processes can be studied in our formalism as well.

We consider a model for gravitational decoherence that has been derived by Kok and Yurtsever [46]. Their model is particularly appealing for our purposes as it leads to a qubit-to-qubit channel, therefore facilitating the analysis. It describes quantum metric fluctuations of qubits that are gravitationally coupled to a background spacetime. The metric is assumed to be described by a quantum state ρ_g that evolves like a regular quantum system. The evolution of the joint system of the metric and the qubit causes the qubit to decohere.

More precisely, let $|0\rangle$, $|1\rangle$ be the energy eigenbasis of the qubit, i.e. we choose the Z-basis to coincide with the eigenbasis of Hamiltonian of the qubit. Then, the free evolution of an energy eigenstate $|k\rangle$ in a quantum metric $|g_j\rangle$ is given by

$$|k\rangle \otimes |g_j\rangle \mapsto e^{i\omega_k \tau_j}|k\rangle \otimes |g_j\rangle\,, \tag{188}$$

where ω_k is the frequency of the energy eigenstate $|k\rangle$ and where $d\tau = dt\sqrt{g_{\mu\nu}\dot{x}^\mu\dot{x}^\nu}$ and $\dot{x}^\mu/dt$. Note that this evolution is not of product form. The evolution (188), followed by a partial trace over the metric, specifies a qubit-to-qubit channel. This channel depends on the state of the metric. Kok and Yurtsever consider three different scenarios, each with a corresponding state of the quantum metric, leading to three different channels. The three different scenarios are:

1. Fluctuations in flat spacetime,
2. Mass fluctuations in black holes,
3. Gravitational waves.

In the following, we restrict ourselves to the scenario of metric fluctuations in flat spacetime, and denote the resulting channel by C_t^{fluc}. Kok and Yurtsever calculate the effect of the channel on a qubit in the state

$$|\psi\rangle = \frac{1}{\sqrt{2}}\left(|0\rangle + e^{i\varphi}|1\rangle\right) . \tag{189}$$

We modify their analysis and consider the action of the channel C_t^{fluc} on one half of a maximally entangled state instead, i.e. we determine the Choi representation

$$\rho_{AB}(C_t^{\text{fluc}}) = (\mathbb{1}_A \otimes C_t^{\text{fluc}})|\Phi^+\rangle\langle\Phi^+|_{AA'} , \tag{190}$$

(c.f. Supplementary Note 4).

For the metric fluctuations in flat spacetime, Kok and Yurtsever consider a uniformly moving qubit with velocity $dx/dt = v$ in the x-direction in a two-dimensional flat Minkowski space. The state of the metric is given by

$$\rho_g = \int d\vec{a} f(\vec{a})|g_{\vec{a}}\rangle\langle g_{\vec{a}}| , \tag{191}$$

where $f(\vec{a})$ determines the quantum fluctuations of the metric around $\vec{a} = 0$, which are assumed to Gaussian. A derivation along the lines of [46] shows that for the resulting channel C_t^{fluc}, the Choi matrix reads

$$\rho_{AB}\left(C_t^{\text{fluc}}\right) = \frac{1}{2}\begin{pmatrix} 1 & 0 & 0 & \eta(t)e^{i(\gamma\Omega t+\delta(t))}e^{-\Gamma(t)^2} \\ 0 & 0 & 0 & 0 \\ 0 & 0 & 0 & 0 \\ \eta^*(t)e^{-i(\gamma\Omega t+\delta(t))}e^{-\Gamma(t)^2} & 0 & 0 & 1 \end{pmatrix} , \tag{192}$$

where

$$\gamma = \sqrt{1-v^2} , \tag{193}$$

$$\eta(t) = 1 + i\left(\frac{1}{2}(1+v^2)\Omega t\sigma^2\right) , \tag{194}$$

$$\Gamma(t) = (1+v^2)\Omega t\sigma , \tag{195}$$

$$\delta(t) = \frac{1}{4}v^2(5+5v^2+11v^4+3v^6)\Omega^3 t^3\sigma^4 , \tag{196}$$

and where $\eta^*(t)$ is the complex conjugate of $\eta(t)$, with the following real parameters:

- the elapsed time t,
- the particle speed v,
- the transition frequency $\Omega = \omega_1 - \omega_0$,
- the variance σ of the fluctuation.

A comparison of (192) with (172) may make the reader wonder whether the decoherence of this channel can be expressed by the decoherence of an appropriately parametrized dephasing channel, i.e. a Pauli channel with $p_x = p_y = 0$ and some $p_x(t) \in [0,1]$ for all $t \in [0,1]$. This is indeed the case. To see that, we express the off-diagonal terms of the Choi matrix (192) in polar form. The factor $e^{-\Gamma(t)^2}$ is already real and the factor $e^{i(\gamma\Omega t+\delta(t))}$ already has polar form. For $\eta(t)$, we get

$$\eta(t) = 1 + i\left(\frac{1}{2}(1+v^2)\Omega t\sigma^2\right) \tag{197}$$

$$= |\eta|\, e^{i\arg(\eta)} \tag{198}$$

$$= \sqrt{1+\frac{1}{4}(1+v^2)^4\Omega^2 t^2\sigma^2} e^{i\arctan\left(\frac{1}{2}(1+v^2)^2\Omega t\sigma^2\right)} . \tag{199}$$

Therefore, in polar form, the the upper off-diagonal element of the matrix (192), which we shall denote by $\kappa(t)$, is given by

$$\kappa(t) := \sqrt{1+\frac{1}{4}(1+v^2)^4\Omega^2 t^2\sigma^2} e^{-\Gamma(t)^2} e^{i\left(\gamma\Omega t+\delta(t)+\arctan\left(\frac{1}{2}(1+v^2)^2\Omega t\sigma^2\right)\right)} . \tag{200}$$

Hence, the off-diagonal terms of the Choi matrix have a phase

$$\alpha(t) = \gamma\Omega t + \delta(t) + \arctan\left(\frac{1}{2}(1+v^2)^2\Omega t\sigma^2\right) . \tag{201}$$

It turns out that the Choi matrix (192) can be expressed as

$$\rho_{AB}\left(C_t^{\text{fluc}}\right) = (\mathbb{1}\otimes U_{-\alpha(t)})\Big((\mathbb{1}\otimes C^{\text{Pauli}}_{(0,0,p_z(t))})|\Phi^+\rangle\langle\Phi^+|\Big)(\mathbb{1}\otimes U_{\alpha(t)}) \tag{202}$$

$$= (\mathbb{1}\otimes U_{-\alpha(t)})\rho_{AB}(C^{\text{Pauli}}_{(0,0,p_z(t))})(\mathbb{1}\otimes U_{\alpha(t)}) \tag{203}$$

where

$$U_{\alpha(t)} = \begin{pmatrix} 1 & 0 \\ 0 & e^{i\alpha(t)} \end{pmatrix} , \tag{204}$$

$$p_z(t) = \frac{1-\sqrt{1+\frac{1}{4}(1+v^2)^4\Omega^2t^2\sigma^2}e^{-\Gamma(t)^2}}{2} . \tag{205}$$

This means that, up to a local unitary, the channel C_t^{fluc} is a dephasing channel with a deformed strength parameter p. This is good news for us, because local unitaries do not change the decoherence quantity. This can be seen as follows. First note that

$$\text{Dec}(A|E)_{\rho(C_t^{\text{fluc}})} = \frac{1}{d_A}2^{H_{\max}(A|B)_{\rho(C_t^{\text{fluc}})}} \tag{206}$$

$$= \frac{1}{d_A}2^{\max_{\sigma_B}\log d_A F^2(\rho_{AB}(C_t^{\text{fluc}}),\pi_A\otimes\sigma_B)} , \tag{207}$$

where we got the first equality from equation (170), and the second equality from the definition of the max-entropy, see equation (32). Furthermore, we have that

$$\max_{\sigma_B}\log d_A F^2(\rho_{AB}(C_t^{\text{fluc}}),\pi_A\otimes\sigma_B) = \max_{\sigma_B}\log d_A F^2((\mathbb{1}\otimes U_{-\alpha(t)})\rho_{AB}(C^{\text{Pauli}}_{(0,0,p_z(t))})(\mathbb{1}\otimes U_{\alpha(t)}),\pi_A\otimes\sigma_B) \tag{208}$$

$$= \max_{\sigma_B}\log d_A F^2(\rho_{AB}(C^{\text{Pauli}}_{(0,0,p_z(t))}),(\mathbb{1}\otimes U_{\alpha(t)})\pi_A\otimes\sigma_B(\mathbb{1}\otimes U_{-\alpha(t)})) \tag{209}$$

$$= \max_{\sigma_B}\log d_A F^2(\rho_{AB}(C^{\text{Pauli}}_{(0,0,p_z(t))}),\pi_A\otimes U_{-\alpha(t)}\sigma_B U_{\alpha(t)}) \tag{210}$$

$$= \max_{\sigma_B}\log d_A F^2(\rho_{AB}(C^{\text{Pauli}}_{(0,0,p_z(t))}),\pi_A\otimes\sigma_B) , \tag{211}$$

where we used equation (203) for the first equality, the invariance of the fidelity under unitaries in the second equality, and the fact that a unitary does not change the maximization over all states σ_B on B for the last equality. Hence, the decoherence quantity for the channel C_t^{fluc} is the same as the decoherence quantity for the channel $C^{\text{Pauli}}_{(0,0,p_z(t))}$,

$$\text{Dec}(A|E)_{\rho(C_t^{\text{fluc}})} = \text{Dec}(A|E)_{\rho(C^{\text{Pauli}}_{(0,0,p_z(t))})} \tag{212}$$

$$= \frac{1}{4}\left(\sqrt{p_z(t)}+\sqrt{1-p_z(t)}\right)^2 \tag{213}$$

$$= \frac{1}{4}\left(\sqrt{\frac{1-\sqrt{1+\frac{1}{4}(1+v^2)^4\Omega^2t^2\sigma^2}e^{-\Gamma(t)^2}}{2}}+\sqrt{\frac{1+\sqrt{1+\frac{1}{4}(1+v^2)^4\Omega^2t^2\sigma^2}e^{-\Gamma(t)^2}}{2}}\right)^2 \tag{214}$$

A plot of the decoherence quantity (214) as a function of the time t is shown in Supplementary Fig. 10.

The decoherence quantity for amplitude damping

$$C_\lambda^{\text{AD}}(\rho) = E_0\rho E_0^\dagger + E_1\rho E_1^\dagger , \tag{215}$$

where

$$E_0 = \begin{pmatrix} 1 & 0 \\ 0 & \sqrt{1-\lambda} \end{pmatrix}, \quad E_1 = \begin{pmatrix} 0 & \sqrt{\lambda} \\ 0 & 0 \end{pmatrix}. \tag{216}$$

Its Choi matrix is given by

$$\rho_{AB}\left(C_\lambda^{\mathrm{AD}}\right) = \frac{1}{2}\begin{pmatrix} 1 & 0 & 0 & \sqrt{1-\lambda} \\ 0 & 0 & 0 & 0 \\ 0 & 0 & \lambda & 0 \\ \sqrt{1-\lambda} & 0 & 0 & 1-\lambda \end{pmatrix}. \tag{217}$$

The conditional max-entropy of (217) can easily be solved using a semidefinite program (SDP) solver software such as SeDuMi and YALMIP for Matlab (see the SDP formulation of the conditional max entropy in Supplementary Note 2). A plot of the decoherence quantity of C_λ^{AD} is shown on the left hand side of Supplementary Fig. 11 (see the graph with $p = 1$) as a function of λ. It achieves a maximal decoherence of 1.

The decoherence quantity for erasure models

The next family of channels that we want to look at is the family of *erasure channels.* It is a family of channels parametrized by a probability $p \in [0,1]$. We denote the erasure channel with parameter p by C_p^{E}. For every such parameter, the channel C_p^{E} is a qubit-to-qutrit channel. The idea is that a qubit is transmitted via this channel, and with probability $1-p$, it is left unchanged, and with probability p it is erased. In the case of an erasure, the receiver is notified about the erasure, which is modelled by saying that the qubit is mapped to the state $|2\rangle\langle 2|$ of the qutrit. We can write this channel as

$$C_p^{\mathrm{E}}(\rho) = (1-p)\widehat{\rho} + p|2\rangle\langle 2|, \tag{218}$$

where $\widehat{\rho}$ is the image of the embedding which maps the qubit onto the subspace of the qutrit spanned by $|0\rangle$ and $|1\rangle$. We get that

$$C_p^{\mathrm{E}}\begin{pmatrix} a & b \\ c & d \end{pmatrix} = \begin{pmatrix} (1-p)a & (1-p)b & 0 \\ (1-p)c & (1-p)d & 0 \\ 0 & 0 & p \end{pmatrix} \tag{219}$$

The Choi matrix of this channel is given by

$$\rho_{AB}\left(C_p^{\mathrm{E}}\right) = \frac{1}{2}\begin{pmatrix} 1-p & 0 & 0 & 0 & 1-p & 0 \\ 0 & 0 & 0 & 0 & 0 & 0 \\ 0 & 0 & p & 0 & 0 & p \\ 0 & 0 & 0 & 0 & 0 & 0 \\ 1-p & 0 & 0 & 0 & 1-p & 0 \\ 0 & 0 & p & 0 & 0 & p \end{pmatrix}. \tag{220}$$

A plot of the decoherence quantity for this channel can be seen on the right hand side of Supplementary Fig. 11.

Supplementary Note 6: An example test for gravitational decoherence

An optomechanical setting and its model for gravitational decoherence

We have seen how to calculate the decoherence quantity for several models of decoherence in the previous section. Now we make things more concrete by devising a full test of a particular model of gravitational decoherence. This includes not only the calculation of the decoherence quantity, but also the minimal CHSH quantity β that one would need to measure in order to falsify the model, and an estimate of the point in time at which it is most likely to measure such a value.

The objective here is to create two entangled photonic qubits in which one photon is prepared in an opto-mechanical system that is itself subject to gravitational decoherence — if there is any — and the other photon is prepared in an identical cavity except the mirrors are fixed and cannot move. This model is a modification of the model first proposed by Bouwmeester [45] in which an itinerant single photon pulse is injected into a cavity rather than created intra-cavity as here. Our modification avoids the problem that the time over which the photons interact with the mechanical element is stochastic and determined by the random times at which the photons enter and exit the cavity through an end mirror. In the new scheme, the cavities are assumed to have almost perfect mirrors — very narrow line width (see for example[47]).

The intracavity single photon Raman source is described in Nisbet-Jones, et al. [48]. In this scheme (see Supplementary Fig. 12) a control pulse can quickly and efficiently prepare a cavity mode in a single photon state by driving a Raman transition between two hyperfine levels we label as $|g\rangle, |e\rangle$. In our scheme there are two optical cavities otherwise identical except in one of the cavities a mechanical element can respond to the radiation pressure force of light.

We will assume that we can prepare the atomic sources in an arbitrary entangled state $|g,e\rangle + |e,g\rangle$, for example, using the trapped ion schemes of Monroe [49]. In addition we will assume that we can make arbitrary rotations in the g, e subspace of each source and also make fast efficient single shot readout of the state of each source, for example using fluorescence shelving. This means we can readout the atomic qubit in each cavity in any basis.

The write laser implements the Hamiltonian $H_w = i\hbar\Omega(t)(a^\dagger|e\rangle\langle g| - a|g\rangle\langle e|)/2$. This is a rotation in the state space $\{|g\rangle|0\rangle, |e\rangle|1\rangle\}$. We can thus prepare arbitrary states of the form $\cos\theta/2|g\rangle|0\rangle + \sin\theta/2|e\rangle|1\rangle$, where θ is determined by the pulse area. We will refer to the case of $\theta = \pi$ as a π-pulse. Note that if the source is in the excited state $|e\rangle$ and the cavity is in the vacuum, no photon is excited.

Starting with the cavities in the vacuum state the protocol proceeds as follows:

1. prepare the source atoms in the state $|g,e\rangle + |e,g\rangle$.
2. apply the write laser with a π-pulse
3. free evolution of the OM systems for a time T
4. apply the write laser with a π-pulse
5. readout the atomic state in each cavity.

At the end of Step 2, the state of the sources and the cavities is $|\psi_2\rangle = |e,e\rangle \otimes (|1,0\rangle + |0,1\rangle)$ where $|n,m\rangle = |n\rangle \otimes |m\rangle$ with each factor being a photon number eigenstate.

Gravitational decoherence

We will use Diosi's theory of gravitational decoherence[50]. This is equivalent to the decoherence model introduced in Kafri et al. [51]. One mirror of the opto-mechanical cavity is free to move in a harmonic potential with frequency ω_m. The master equation for a massive particle moving in a harmonic potential, including gravitational decoherence is

$$\frac{d\rho}{dt} = -i\omega_m[b^\dagger b, \rho] - \Lambda_{\text{grav}}[b + b^\dagger, [b + b^\dagger, \rho]] \tag{221}$$

where

$$b = \sqrt{\frac{m\omega_m}{2\hbar}}\hat{x} + i\frac{1}{\sqrt{2\hbar m\omega_m}}\hat{p} \tag{222}$$

with $\hat{x}, \hat{p}$ the usual canonical position and momentum operators. The gravitational decoherence rate Λ_{grav} is given by

$$\Lambda_{\text{grav}} = \frac{2\pi}{3}\frac{G\Delta}{\omega_m} \tag{223}$$

with G the Newton gravitational constant and Δ the density of the mechanical element. As one might expect Λ_{grav} is quite small, of the order of 10^{-8} s^{-1} for suspended mirrors (as in LIGO) with $\omega_m \sim 1$.

Form a phenomenological perspective the effect of gravitational decoherence is analogous to a Browning heating effect. To see this we note that the average vibrational quantum number increases diffusively

$$\frac{d\langle b^\dagger b\rangle}{dt} = 2\Lambda_{\text{grav}} \tag{224}$$

Indeed, one could simulate this effect by adding a stochastic driving force to the mechanical element via the stochastic Hamiltonian

$$H_s = \frac{dI}{dt}(b + b^\dagger) \tag{225}$$

where $I(t)$ satisfies an Ito stochastic differential equation,

$$dI(t) = \sqrt{4\Lambda_{\text{grav}}}\, dW(t) \tag{226}$$

where $dW(t)$ is the Weiner increment. Averaging over all histories of the stochastic driving force gives the final term in Eq. 221.

In the absence of mechanical dissipation, there is no steady state. In reality the mechanical quality factor, $Q = \omega_m/\gamma_m$, is finite leading to a steady state with mean phonon number given by

$$\langle b^\dagger b\rangle_{ss} = \frac{2\Lambda_{\text{grav}}}{\gamma_m} \tag{227}$$

This of course assumes that there is no additional mechanical heating (regular thermodynamic kind): hardly a realistic assumption. This adds a large (comparatively) additional term to Λ_{grav} so that we find (for $k_B T >> \hbar\omega_m$),

$$\Lambda_{\text{grav}} \to \Lambda_{\text{grav}} + \Lambda_{\text{heat}}\ , \quad \text{where } \Lambda_{\text{heat}} = \frac{k_B T}{\hbar Q}\ . \tag{228}$$

Given the incredibly large quality factor of $Q = 10^{10}$, one would need to cool the mechanical element to nano-Kelvin for the thermodynamical heating to be of the order of the gravitational heating.

Optomechanical probe of gravitational decoherence.

The optomechanical Hamiltonian in cavity-one is

$$H_{\text{om}} = \hbar\omega_m b^\dagger b + \hbar g_0(b + b^\dagger) \tag{229}$$

g_0 is the single photon optomechanical coupling rate. Typically $g_0 \sim 1\ \text{s}^{-1}$ for the sorts of cavities we are considering here. This is about the same order of magnitude as ω_m. In new field OM cavity technologies, g_0 can be as high as $10^3\ \text{s}^{-1}$ however in such cases the mechanical frequency is also typically much higher $\sim$ tens of MHz. The interaction time is T which is short compared to the cavity decay time (which we neglect). We will assume that the mechanics starts in a thermal state, the steady state of the system subject to gravitational decoherence, heating and dissipation. This is given by

$$\rho_{\text{om}} = \frac{1}{\pi\bar{n}}\int d^2\alpha\ e^{-\frac{|\alpha|^2}{\bar{n}}}\ |\alpha\rangle_b\langle\alpha| \tag{230}$$

where $\bar{n} = \langle b^\dagger b\rangle_{ss}$ is the steady state mean phonon number given in Eq. 227.

It is simplest to work in an interaction picture defined by the mechanical free dynamics,

$$H_{\text{om,I}} = \hbar g_0(b e^{-i\omega_m t} + b^\dagger e^{i\omega_m t}) \tag{231}$$

The corresponding unitary evolution operator is

$$U(t) = e^{\beta(t)b^\dagger - \beta^*(t)b} \tag{232}$$

where

$$\beta(t) = \frac{g_0}{\omega_m}(e^{-i\omega_m t} - 1) \tag{233}$$

The initial state for the OM interaction is the state at the end of Step 2

$$\rho_{\text{om}}(0) = \frac{1}{2}\left(|1,0\rangle\langle 1,0| + |0,1\rangle\langle 0,1| + |1,0\rangle\langle 0,1| + |0,1\rangle\langle 1,0|\right) \otimes \rho_m \tag{234}$$

where ρ_m is the state of the mechanical element at the start of the protocol, a thermal state. We can ignore the state of the atomic sources at this stage as they do not participate in the OM interaction.

The state of the optomechanical system after an interaction time T is given by

$$\rho_{\text{om}}(t) = \frac{1}{2}\left(|1,0\rangle\langle 1,0|\rho_m + |0,1\rangle\langle 0,1|U(t)\rho_m U^\dagger(t) + |1,0\rangle\langle 0,1|\rho_m U^\dagger(t) + |0,1\rangle\langle 1,0|U(t)\rho_m\right) \tag{235}$$

The reduced state of the cavity fields is given by tracing out the mechanical degree of freedom,

$$\rho_f(t) = \frac{1}{2}\left(|1,0\rangle\langle 1,0| + |0,1\rangle\langle 0,1| + R^*|1,0\rangle\langle 0,1| + R|0,1\rangle\langle 1,0|\right) \tag{236}$$

where

$$R = e^{-(1+2\bar{n})|\beta(t)|^2/2} \tag{237}$$

where

$$\bar{n} = \overline{n}_{\text{grav}} + \overline{n}_{\text{heat}} \tag{238}$$

$$:= \frac{2\Lambda_{\text{grav}}}{\gamma_m} + \frac{2\Lambda_{\text{heat}}}{\gamma_m} \tag{239}$$

with (as above)

$$\Lambda_{\text{grav}} = \frac{2\pi}{3}\frac{G\Delta}{\omega_m}, \quad \Lambda_{\text{heat}} = \frac{k_B T}{\hbar Q} \tag{240}$$

and

$$|\beta(t)|^2 = \frac{4g_0^2}{\omega_m^2}\sin^2(\omega_m t/2) \tag{241}$$

Continuing with the protocol from Step 4, now results in the state of the atom-field system

$$\rho_{af}(t) = \rho_a(t) \otimes |00\rangle\langle 00| \tag{242}$$

where

$$\rho_a(t) = \frac{1}{2}(|ge\rangle\langle ge| + |eg\rangle\langle eg| + R^*|eg\rangle\langle ge| + R|ge\rangle\langle eg|) \tag{243}$$

The suppression of coherence due to the thermal state of the mechanics has been transferred to a reduction of entanglement in the atomic sources. A readout of the atomic sources will reveal this through either state tomography or via a reduction in a CHSH correlation for a Bell-type experiment.

The function $R(t)$ is a periodic function of time. At each period of the motion it returns to its initial value of zero and the cavity field state would return to the fully entangled state it was in after Step 2. If we chose $T = 2\pi/\omega_m$ then the protocol will return the atomic system to the same entangled state in which it began. This is because we have ignored the heating of the mechanics over the period T so the only way decoherence enters is through the initial thermal excitation of the mechanics. In effect the protocol is a thermometer. We thus see that for maximum effect we need to ensure $g_0 \gg \omega_m$. On the other hand, gravitational heating requires a small value of ω_m and typically such OM systems have $g_0/\omega_m \ll 1$. Perhaps technical advances will enable OM systems with long mechanical periods and large single photon coupling. Of course this will also require sub hertz cavity line widths. In the (exceptionally) optimistic case we can take $T \sim 1$ nK, $\omega_m \sim 1$ s^{-1}, $\gamma_m \sim 10^{-10}$ s^{-1} so that $Q \sim 10^{10}$.

An experimental test of the model

In the previous subsection, an optomechanical setting has been described. Making some assumptions about how gravitational decoherence influences the optomechanical system, a model has been given that describes how the state of the optomechanical system changes over time. In this section, we consider this model for the state of the optomechanical system as given and analyze it using our decoherence test formalism. We calculate the amount of decoherence that would be introduced to the optomechanical system if the model was correct. We compare this

to the amount of decoherence that one would observe if there was no such gravitational decoherence, determining the difference between the two predictions. We devise an experiment that aims at estimating the actual amount of decoherence at a point in time when this difference is maximal. This turns the optomechanical experiment into a test that allows to falsify the above model for gravitational decoherence if it was wrong. This shows that the decoherence testing formalism presented in this work can be applied in situations where the physical process is unknown. It allows to subject proposed models of the process to a consistency check.

We first present the predicted values of $\mathrm{Dec}(A|E)_\rho$ of the optomechanical system for the two cases where gravitational decoherence is present or absent, respectively, for some example parameters of the experiment. We then calculate the CHSH value β that one would have to measure in order to falsify the model for gravitational decoherence.

The main quantity of interest in our analysis is the decoherence quantity $\mathrm{Dec}(A|E)_\rho$ for the state $\rho_{AB} = \rho_f(t)$ described in equation (236). The calculation of the decoherence quantity for this state is very simple using the tools that we developped in Supplementary Note 5. To see this, we make a change of basis for Bob, swapping the two basis vectors, i.e. $|0\rangle \mapsto |1\rangle$, $|1\rangle \mapsto |0\rangle$. With this change of basis, the density matrix reads:

$$\rho_f(t) = \frac{1}{2}\begin{pmatrix} 1 & 0 & 0 & R \\ 0 & 0 & 0 & 0 \\ 0 & 0 & 0 & 0 \\ R & 0 & 0 & 1 \end{pmatrix} \tag{244}$$

(note that R is real). A comparison of (244) with (172) reveals that this is a Pauli channel with $p_x = p_y = 0$ and $p_z = (1-R)/2$. Recall from Supplementary Note 5 that this is a dephasing channel. According to equation (183), its decoherence is given by

$$\mathrm{Dec}(A|E)_\rho = \frac{1}{4}\left(\sqrt{\frac{1-R}{2}} + \sqrt{1 - \frac{1-R}{2}}\right) \tag{245}$$

$$= \frac{1}{4}\left(1 + \sqrt{1-R^2}\right) \tag{246}$$

$$= \frac{1}{4}\left(1 + \sqrt{1 - \exp\left(-4(1+2\overline{n})\frac{g_0^2}{\omega_m^2}\sin^2\left(\frac{\omega_m t}{2}\right)\right)}\right). \tag{247}$$

If the above model is correct and gravitational decoherence occurs, both the gravitational interaction and the mechanical heating contribute to the average vibrational quantum number, i.e. we have

$$\overline{n} = \overline{n}_{\mathrm{grav}} + \overline{n}_{\mathrm{heat}} \tag{248}$$

$$= \frac{4\pi G}{3}\frac{1}{\gamma_m \omega_m}\Delta + \frac{2k_B}{\hbar}\frac{1}{\omega_m}T \tag{249}$$

and thus

$$\mathrm{Dec}(A|E)_\rho = \frac{1}{4}\left(1 + \sqrt{1 - \exp\left(-4\left(1 + 2\left(\frac{4\pi G}{3}\frac{1}{\gamma_m\omega_m}\Delta + \frac{2k_B}{\hbar}\frac{1}{\omega_m}T\right)\right)\frac{g_0^2}{\omega_m^2}\sin^2\left(\frac{\omega_m t}{2}\right)\right)}\right) \quad \text{(gr. dec. included).} \tag{250}$$

If gravitational decoherence is absent, then only the mechanical heating contributes to the average vibrational quantum number, i.e. we have

$$\overline{n} = \overline{n}_{\mathrm{heat}} \tag{251}$$

$$= \frac{2k_B}{\hbar}\frac{1}{\omega_m}T \tag{252}$$

and thus

$$\mathrm{Dec}(A|E)_\rho = \frac{1}{4}\left(1 + \sqrt{1 - \exp\left(-4\left(1 + 2\left(\frac{2k_B}{\hbar}\frac{1}{\omega_m}T\right)\right)\frac{g_0^2}{\omega_m^2}\sin^2\left(\frac{\omega_m t}{2}\right)\right)}\right) \quad \text{(gr. dec. neglected).} \tag{253}$$

Supplementary Fig. 13 shows how the decoherence quantity in equation (250) as a function of time varies for different materials of the mechanical element and different temperatures, compared to the case where there is no gravitational decoherence as in equation (253).

In order to rule out the model for gravitational decoherence, one needs to measure a CHSH value β which is incompatible with the value of $\mathrm{Dec}(A|E)_\rho$ given in (250). The minimal value β_{fals} of β that needs to be measured for this falsification can be calculated using Theorem 1: Using MATLAB, we numerically evaluated the quantum bound on $\mathrm{Dec}(A|E)_\rho$, which is given as a point-wise maximization problem in Theorem 1. We inverted the resulting set of data points and interpolated a function from the resulting data using Mathematica. The resulting function takes a value of $\mathrm{Dec}(A|E)_\rho$ as its input and outputs the minimal β that needs to be exceeded in a measurement in order to rule out the given value of $\mathrm{Dec}(A|E)_\rho$. Thus, applying this function to the curves of the $\mathrm{Dec}(A|E)_\rho$ values of the gravitational decoherence model in Supplementary Fig. 13 yields the curves for β_{fals}. The results are plotted in Supplementary Fig. 14 for the same materials and temperatures as above.

In order to determine whether it is promising to measure a value of β that lies above β_{fals}, we need to determine the value β_{mech} of β which is predicted in the case where gravitational decoherence is absent, i.e. where we only have mechanical heating. We can do that exactly: Equation (236) gives us an expression for the state, which we consider for the value of R given in the case of mechanical heating only, $\overline{n} = \overline{n}_{\mathrm{mech}}$. Then we calculate the value of β_{mech} for the case where the measurements are taken to be the standard CHSH measurements

$$A_0 = \sigma_x \,, \qquad A_1 = \sigma_z \,,$$
$$B_0 = \frac{\sigma_x - \sigma_z}{\sqrt{2}} \,, \qquad B_1 = \frac{\sigma_x + \sigma_z}{\sqrt{2}} \,,$$

where σ_x, σ_z are the Pauli x- and z-operator, respectively. The resulting curves are shown in Supplementary Fig. 14 as solid curves. It turns out that for the relevant time interval (where β_{mech} is larger than either of the β_{fals}), the curve of β_{mech} for the standard CHSH measurements is almost identical to the curve one would get for the optimal measurements for each time t. The latter can be calculated using a formula presented in [52]. This is an experimentally desirable fact: Using a fixed measurement independent of the measurement time is almost optimal.

The most promising measurement time for a falsification of the gravitational decoherence model is given by the time when β_{mech} (that one may hope to actually measure) is high but β_{fals} (which one needs to exceed) is low. Thus, the optimal measurement time can be calculated as the time t_{max} that maximizes the gap function

$$g(t) := \beta_{\mathrm{mech}}(t) - \beta_{\mathrm{fals}}(t) \,. \tag{254}$$

This gap function is depends on the density Δ of the mechanical element and its temperature T. One can see that temperatures that look promising for a falsification measurement when looking at the $\mathrm{Dec}(A|E)_\rho$ values in Supplementary Fig. 13 turn out to be too warm when looking at the experimentally relevant analysis of the β values in Supplementary Fig. 14. As an example, we have calculated the optimal measurement times for $T = 1$ nK for the densities of aluminum and rhenium. They are visualized in Supplementary Fig. 15. If there is no gravitational decoherence, one needs to measure values of β that are ~ 0.1 close (aluminum) or ~ 0.2 close (rhenium) to the value that one can maximally measure using the standard CHSH measurements, in order to exclude gravitational decoherence.

Supplementary References

[1] Wilde, M. *Quantum Information Theory* (Cambridge University Press, 2013).

[2] Barnum, H., Knill, E. & Nielsen, M. A. On quantum fidelities and channel capacities. *IEEE Transactions on Information Theory* **46**, 1317–1329 (2000).

[3] Stinespring, W. F. Positive Functions on C*-algebras. *Proc. Amer. Math. Soc.* **6** (1955).

[4] Schumacher, B. & Nielsen, M. A. Quantum data processing and error correction. *Phys. Rev. A* **54**, 2629–2635 (1996).

[5] Lloyd, S. Capacity of the noisy quantum channel. *Phys. Rev. A* **55**, 1613–1622 (1997).

[6] Shor, P. The quantum channel capacity and coherent information. Lecture notes at `http://www.msri.org/publications/ln/msri/2002/quantumcrypto/shor/1/` (2002)

[7] Devetak, I. The private classical capacity and quantum capacity of a quantum channel. *IEEE Trans. Inf. Theory* **51**, 44–55 (2005).

[8] Renner, R. *Security of Quantum Key Distribution.* Ph.D. thesis, ETH Zürich (2005).

[9] Dupuis, F., Berta, M., Wullschleger, J. & Renner, R. One-shot decoupling. *Communications in Mathematical Physics* **328**, 251–284 (2014).

[10] Hayden, P., Horodecki, M., Yard, J. & Winter, A. A decoupling approach to the quantum capacity. *Open Systems and Information Dynamics* **15**, 7–19 (2008).

[11] König, R., Renner, R. & Schaffner, C. The operational meaning of min- and max-entropy. *IEEE Trans. Inf. Theory* **55**, 4337–4347 (2009).

[12] Tomamichel, M., Renner, R. & Colbeck, R. A fully quantum asymptotic equipartition property. *IEEE Trans. Inf. Theory* **55**, 5840–5847 (2009).

[13] Clauser, J. F., Horne, M. A., Shimony, A. & Holt, R. A. Proposed experiment to test local hidden-variable theories. *Phys. Rev. Lett.* **23**, 880–884 (1969).

[14] Scarani, V. & Gisin, N. Quantum communication between N partners and bell's inequalities. *Phys. Rev. Lett.* **87**, 117901 (2001).

[15] Horodecki, R. & Horodecki, M. Information-theoretic aspects of inseparability of mixed states. *Phys. Rev. A* **54**, 1838–1843 (1996).

[16] Horodecki, R., Horodecki, P. & Horodecki, M. Violating Bell inequality by mixed spin-1/2 states: necessary and sufficient condition. *Phys. Lett. A* **200**, 340–344 (1995).

[17] Tomamichel, M. *A Framework for Non-Asymptotic Quantum Information Theory.* Ph.D. thesis, ETH Zürich (2012).

[18] Vitanov, A., Dupuis, F., Tomamichel, M. & Renner, R. Chain Rules for Smooth Min- and Max-Entropies. *IEEE Trans. Inf. Theory* **59**, 2603–2612 (2013).

[19] Acín, A. *et al.* Device-independent security of quantum cryptography against collective attacks. *Phys. Rev. Lett.* **98**, 230501 (2007).

[20] Mackey, G. W. *The mathematical foundations of quantum mechanics* (W.A. Benjamin, 1963).

[21] Edwards, C. The operational approach to algeraic quantum theory i. *Comm. Math. Phys.* **16**, 207–230 (1970).

[22] Davies, E. B. & Lewis, J. T. An operational approach to quantum probability. *Commun. Math. Phys.* **17**, 239–260 (1970).

[23] Hardy, L. Quantum theory from five reasonable axioms. Preprint at `http://arxiv.org/abs/quant-ph/0101012` (2001)

[24] Masanes, L. & Müller, M. P. A derivation of quantum theory from physical requirements. *New J. Phys.* **13**, 063001 (2011).

[25] Masanes, L., Müller, M. P., Augusiak, R. & Pérez-García, D. Existence of an information unit as a postulate of quantum theory. *Proceedings of the National Academy of Sciences* **110**, 16373–16377 (2013).

[26] Chiribella, G., D'Ariano, G. M. & Perinotti, P. Informational derivation of quantum theory. *Phys. Rev. A* **84**, 012311 (2011).

[27] Dakic, B. & Brukner, C. Quantum theory and beyond: Is entanglement special? In Halvorson, H. (ed.) *Deep Beauty: Understanding the Quantum World through Mathematical Innovation*, 365–392 (Cambridge University Press, 2011).

[28] Ududec, C. *Perspectives on the Formalism of Quantum Theory.* Ph.D. thesis, University of Waterloo (2012).

[29] Pfister, C. & Wehner, S. An information-theoretic principle implies that any discrete physical theory is classical. *Nat. Commun.* **4**, 1851 (2013).

[30] Barrett, J. Information processing in generalized probabilistic theories. *Phys. Rev. A* **75**, 032304 (2007).

[31] Barnum, H. & Wilce, A. Ordered linear spaces and categories as frameworks for information-processing characterizations of quantum and classical theory. Preprint at `http://arXiv.org/abs/0908.2354` (2009).

[32] Barnum, H., Barrett, J., Leifer, M. & Wilce, A. Teleportation in general probabilistic theories. Preprint at `http://arXiv.org/abs/0805.3553` (2008).

[33] Barnum, H., Gaebler, C. P. & Wilce, A. Ensemble steering, weak self-duality, and the structure of probabilistic theories. *Foundations of Physics* **43**, 1411–1427 (2013).

[34] Barnum, H. & Wilce, A. Information processing in convex operational theories. *Electron. Notes Theor. Comput. Sci.* **270**, 3–15 (2011).

[35] Pfister, C. One simple postulate implies that every polytopic state space is classical. Preprint at `http://arXiv.org/abs/1203.5622` (2012).

[36] Janotta, P. & Lal, R. Generalized probabilistic theories without the no-restriction hypothesis. *Phys. Rev. A* **87**, 052131 (2013).

[37] Nielsen, M. A. & Chuang, I. L. *Quantum Computation and Quantum Information* (Cambridge University Press, 2000).

[38] Summers, S. J. & Werner, R. Bell's inequalities and quantum field theory. i. general setting. *J. Math. Phys.* **28**, 2440

(1987).
[39] Scholz, V. B. & Werner, R. F. Tsirelson's problem. Preprint at `http://arxiv.org/abs/0812.4305` (2008).
[40] Doherty, A. C., Liang, Y.-C., Toner, B. & Wehner, S. The quantum moment problem and bounds on entangled multi-prover games. *Proc. 23rd IEEE Conf. on Computational Complexity (CCC'08)* 199–210 (2008).
[41] Kraft, C. H. Some conditions for consistency and uniform consistency of statistical procedures. In *University of California Publications in Statistics, Vol. 1*, 125–142 (University of California Press, 1955).
[42] Fuchs, C. A. & van de Graaf, J. Cryptographic distinguishability measures for quantum-mechanical states. *IEEE Trans. Inf. Theory* **45**, 1216–1227 (1999).
[43] Barrett, J. *et al.* Nonlocal correlations as an information-theoretic resource. *Phys. Rev. A* **71**, 022101 (2005).
[44] Toner, B. Monogamy of non-local quantum correlations. *Proc. R. Soc. A* **465**, 59–69 (2009).
[45] Marshall, W., Simon, C., Penrose, R. & Bouwmeester, D. Towards quantum superpositions of a mirror. *Phys. Rev. Lett.* **91**, 130401 (2003).
[46] Kok, P. & Yurtsever, U. Gravitational decoherence. *Phys. Rev. D* **68**, 085006 (2003).
[47] Kessler, T. *et al.* A sub-40-mhz-linewidth laser based on a silicon single-crystal optical cavity. *Nature Photonics* **6**, 687 (2012).
[48] Nisbet-Jones, B. R., Dilley, J., Ljunggren, D. & Kuhn, A. Highly efficient source for indistinguishable single photons of controlled shape. *New J. Phys.* **13**, 103036 (2011).
[49] Duan, L.-M. & Monroe, C. Colloquium: Quantum networks with trapped ions. *Rev. Mod. Phys.* **82**, 1209–1224 (2010).
[50] Diósi, L. Models for universal reduction of macroscopic quantum fluctuations. *Phys. Rev. A* **40**, 1165–1174 (1989).
[51] Kafri, D., Taylor, J. M. & Milburn, G. J. A classical channel model for gravitational decoherence. *New J. Phys.* **16**, 065020 (2014).
[52] Horodecki, R., Horodecki, P. & Horodecki, M. Violating Bell inequality by mixed spin-1/2 states: necessary and sufficient condition. *Phys. Lett. A* **200**, 340–344 (1995).
